# Multi-tensor fixel-based metrics in tractometry: application to multiple sclerosis

**DOI:** 10.3389/fnins.2024.1467786

**Published:** 2024-12-20

**Authors:** Erick Hernandez-Gutierrez, Ricardo Coronado-Leija, Manon Edde, Matthieu Dumont, Jean-Christophe Houde, Muhamed Barakovic, Stefano Magon, Alonso Ramirez-Manzanares, Maxime Descoteaux

**Affiliations:** ^1^Sherbrooke Connectivity Imaging Lab (SCIL), Computer Science Department, University of Sherbrooke, Sherbrooke, QC, Canada; ^2^Bernard and Irene Schwartz Center for Biomedical Imaging, Department of Radiology, New York University School of Medicine (NYU), New York, NY, United States; ^3^Imeka Solutions Inc., Sherbrooke, QC, Canada; ^4^Pharma Research and Early Development, Neuroscience and Rare Diseases Roche Innovation Center Basel, F. Hoffmann-La Roche Ltd., Basel, Switzerland; ^5^Computer Science Department, Centro de Investigación en Matemáticas A.C. (CIMAT), Guanajuato, Mexico

**Keywords:** diffusion MRI, tractometry, fixel-based analysis, multi-tensor model, multiple sclerosis

## Abstract

Traditional Diffusion Tensor Imaging (DTI) metrics are affected by crossing fibers and lesions. Most of the previous tractometry works use the single diffusion tensor, which leads to limited sensitivity and challenging interpretation of the results in crossing fiber regions. In this work, we propose a tractometry pipeline that combines white matter tractography with multi-tensor fixel-based metrics. These multi-tensors are estimated using the stable, accurate and robust to noise Multi-Resolution Discrete Search method (MRDS). The spatial coherence of the multi-tensor field estimated with MRDS, which includes up to three anisotropic and one isotropic tensors, is tractography-regularized using the Track Orientation Density Imaging method. Our end-to-end tractometry pipeline goes from raw data to track-specific multi-tensor-metrics tract profiles that are robust to noise and crossing fibers. A comprehensive evaluation conducted in a phantom simulating healthy and damaged tissue with the standard model, as well as in a healthy cohort of 20 individuals scanned along 5 time points, demonstrates the advantages of using multi-tensor metrics over traditional single-tensor metrics in tractometry. Qualitative assessment in a cohort of patients with relapsing-remitting multiple sclerosis reveals that the pipeline effectively detects white matter anomalies in the presence of crossing fibers and lesions.

## 1 Introduction

Advancements in diffusion magnetic resonance imaging (dMRI) have facilitated our understanding of the brain's intricate architecture and organization (Le Bihan, 2003). By measuring the diffusion of water molecules within the brain tissue, dMRI provides valuable information to investigate the connectivity and assess the white matter (WM) microstructure of pathways in the brain. Voxels in the WM can contain different axonal fiber populations with complex configurations (Jeurissen et al., 2013). Each one of these populations is called *fixel*, which denotes the discrete component of a *fiber element* (Raffelt et al., 2015; Tournier et al., 2019). Fixels and their properties, like orientation and tissue metrics, are fully determined by the voxel in which they reside. Local modeling allows for estimating these fixel properties at each voxel of the dMRI data (Alexander et al., 2017; Jelescu and Budde, 2017). Tractography can use these locally estimated fixel orientations to reconstructs the trajectories of the WM, which are often called *streamlines* (Behrens et al., 2007; Jeurissen et al., 2017). Additionally, tractometry (Jones et al., 2005; Yeatman et al., 2012) has emerged as a useful method for quantitative analysis of the WM pathways. It encompasses the streamlines obtained with tractography at the macroscopic level with the metrics obtained from a local modeling method at the microscopic level. This combination enables the analysis of microstructural changes by extracting quantitative metrics along specific WM anatomical tracts. Tractometry insights could potentially serve as a valuable tool for investigating WM characterization and degeneration associated with neurological disorders, such as multiple sclerosis (MS) (Winter et al., 2021; Beaudoin et al., 2021), Alzheimer's disease (Lee et al., 2015), and traumatic brain injury (Huang et al., 2022), among others.

Diffusion Tensor Imaging (DTI) (Basser et al., 1994) is a single fiber method traditionally used to estimate properties of the fixels, averaging the diffusion properties of all the fixels within a voxel. Thus, DTI results in a loss of important information of the fixels, especially when different fiber populations with different properties or lesions are present within the same voxel. This presents an important problem in estimation because WM tissue contains between 66% to 90% of voxels with crossing fibers (Descoteaux, 2008; Jeurissen et al., 2013; Schilling et al., 2017). DTI Limitations motivated the development of more advanced acquisition and local modeling techniques. Multi-shell **H**igh **A**ngular **R**esolution **D**iffusion **I**maging (HARDI) (Tuch et al., 2002) was originally developed to provide anisotropy measures beyond DTI metrics (Tournier et al., 2011) that are more robust to crossing fibers and sensitive to WM alterations, making also tractography more robust (Descoteaux, 2015). HARDI allowed to develop techniques that estimate multiple fixels within a voxel. Notable examples of these techniques are: **Q**-**b**all **I**maging (QBI) (Tuch, 2004; Descoteaux et al., 2007), the **M**ulti-**T**ensor **M**odel (MTM) (Tuch et al., 2002), and **C**onstrained **S**pherical **D**econvolution (CSD) (Tournier et al., 2007). In particular, MTM is a straightforward extension of DTI that represents each one of the fiber populations in the voxel by a different diffusion tensor. However, the estimation of MTM parameters is an ill-posed challenging problem, that requires very high SNR data and large computational resources, restricting it's routine clinical use.

The dMRI signal arising from the WM is composed of several compartments. Thus, taking advantage of HARDI, several techniques were developed to decompose the dMRI data into contributions from various compartments. An example of these multi-compartment methods procedures is the model in Novikov et al. (2019), which depicts the dMRI data as a combination of **I**ntra-**C**ellular (IC), **E**xtra-**C**ellular (EC) and **ISO**tropic (ISO) contributions. Other hybrid methods are based in the MTM like the **F**ree-**W**ater DTI (FW-DTI) (Pasternak et al., 2009), which fits for each voxel a bi-tensor model including an anisotropic tensor for tissue compartment and an isotropic tensor for a free water compartment. The **DI**stribution of **A**nisotropic **M**icr**O**-structural e**N**vironments with **D**iffusion-weighted imaging (DIAMOND) (Scherrer et al., 2015) and the **M**ulti-**R**esolution **D**iscrete-**S**earch (MRDS) (Coronado-Leija et al., 2017) are more general MTM-based methods, which fits up to three restricted anisotropic tensors for the restricted and hindered diffusion compartments and one isotropic tensor for the free diffusion compartment.

**Figure 1:**
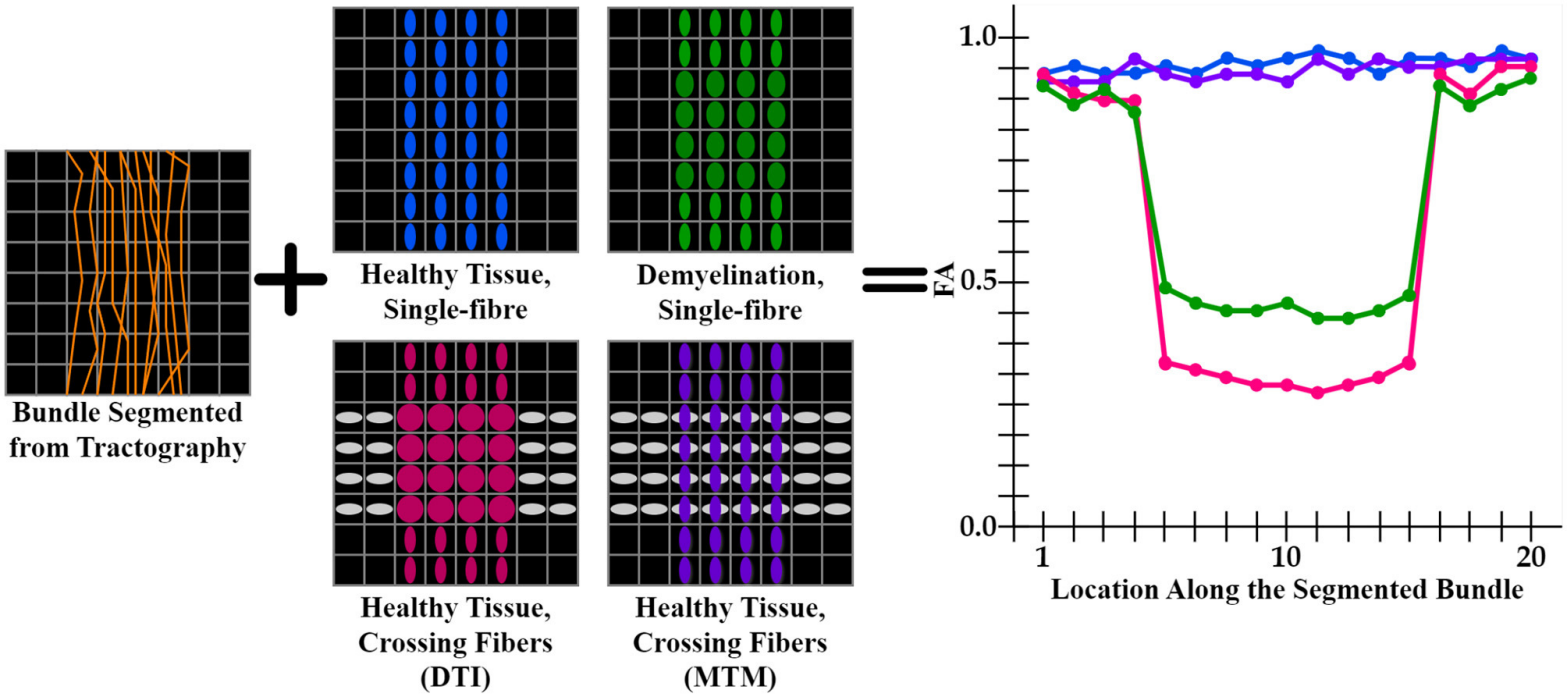
Illustration of an FA tract profile in 4 different scenarios showing the limitation of DTI-based tractometry. All FA tract profiles are generated through the same bundle (orange streamlines). Blue, green, pink tensor fields exhibit a single fixel in each voxel estimated with DTI, while purple tensor field exhibits a multi-fixel estimation with MTM. (blue tensors) A control case with healthy tissue and only one fiber population. This scenario is expected to have high FA values in the tract profile (blue curve). (green tensors) A single fiber with demyelination resulted in an increased RD and decreased FA (green curve). (gray and pink tensors) Tensors are estimated using DTI, where FA is affected in the intersection as oblate tensors are obtained (pink curve). (gray and purple tensors) Tensors are estimated using MTM, which can estimate a different tensor for each fixel at the intersection. This case is expected to have normal FA values as the tractometry only considers FA values of the tensors aligned with the streamlines (purple curve). From this scheme, it is clear that a single-fixel analysis is limited when differentiating between demyelination and crossing fibers based only on the alterations in the tract profiles.

**Figure 2:**
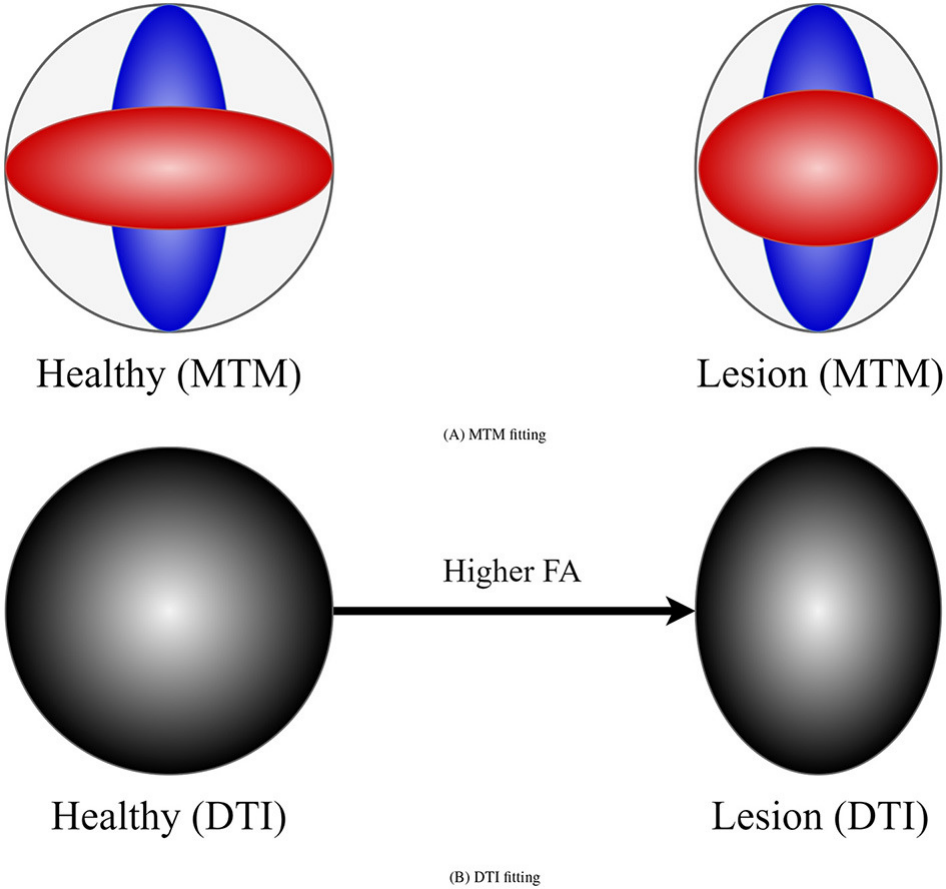
Example of how DTI can lead to counter-intuitive results in the presence of a lesion and crossing fibers. **(A)** Represents a MTM fitting showing the presence of crossing fibers in two scenarios: healthy (left) and damaged (right) tissue. Left scenario exhibits two healthy fiber populations. Right scenario shows a healthy fiber population and another with lesion. **(B)** Represents a tensor obtained with DTI in the healthy and damaged scenarios. In the presence of lesion DTI shows a sharper tensor, leading to a higher FA compared with the healthy scenario. This is a consequence of a lesion only in one of the underlying fiber populations for which DTI is not sensitive.

DTI (Basser et al., 1994) metrics are the most widely used metrics for tractometry (Jones et al., 2005). Although DTI metrics have the potential to be biomarkers, they have inconsistent sensitivity to characterize the WM as they are easily biased. For example, the common **F**ractional **A**nisotropy (FA) metric is informative about changes in WM microstructure caused by pathology, but crossing fibers bias it. FA decreases in fiber crossing voxels because oblate tensors are obtained, which leads to an alteration in the resulting FA tract profile in the tractometry as shown in Figure 1. These alterations can be confused with alterations derived from WM degeneration, which is also illustrated in Figure 1, leading to erroneous or ambiguous interpretations. Moreover, in the presence of crossing fibers together with pathology, FA increases, which could seem counterintuitive. However, this could happen, when only one of the fiber populations in the crossing is affected by the pathology, then the resulting single-tensor may become sharper, see Figure 2. Approaches that have studied other DTI metrics, like the radial diffusivity (RD) metric, have shown that RD is a promising biomarker for demyelination (Song et al., 2002, 2005; de Vries, 2010). However, they have reported that RD can be inconsistent, presenting challenges in its reliability and reproducibility and resulting in misleading results. Besides, co-existing inflammation, edema, and crossing fibers can significantly impact on the DTI metrics at the same time (Ye et al., 2020).

Multi-fixel methods have further expanded the scope of tractometry, resulting in tract-specific analyses less impacted by crossing fibers. Remarkable examples are the Automated Fiber-tract Quantification (Yeatman et al., 2012; Kruper et al., 2021), the Connectivity-based Fixel Enhancement (Raffelt et al., 2015), the Fixel-Based Analysis framework (Dhollander et al., 2021), the Tractometry_flow pipeline (Cousineau et al., 2017; Kurtzer et al., 2017; Di Tommaso et al., 2017) and, recently, the *UNRAVEL* framework (Delinte et al., 2023). Other tractometry frameworks have combined DTI metrics with other metrics including fixel-based metrics like the **A**pparent **F**iber **D**ensity (AFD). For example, the framework called *Profilometry* (Dayan et al., 2015) performs a simultaneous analysis of DTI metrics and other metrics, resulting in tract profiles as parameterized curves in a multi-dimensional space. Nonetheless, the crossing fibers bias in DTI metrics still limits it. Besides, these types of multi-fixel methods face several challenges and limitations. As example, frameworks informed with CSD metrics such as AFD, while sensitive, do not have a straightforward biological interpretation; moreover, they could be biased as CSD employ a fixed response function across the entire WM (Dell'Acqua et al., 2007; Jones et al., 2013). On the other hand, previous tractometry results using MTM fixel-based metrics are not free of limitations. For instance, they need more complex multi-shell dMRI acquisitions (Scherrer and Warfield, 2012) and are limited to a maximum of 2 fixels per voxel (Delinte et al., 2023). This is insufficient in many brain regions, e.g. the centrum semiovale, where 3 fiber populations cross from the corticospinal tract, corpus callosum, and superior longitudinal tract intersect. Additionally, fixel-FA estimation has shown to be affected by high levels of noise and inconsistent through scan-rescan experiments (Mishra et al., 2014) as a consequence of MTM fitting being numerically unstable (Tuch et al., 2002). MTM-based methods generally struggle to accurately estimate the required number of tensors per voxel (*N*). These methods tend to overestimate the value of *N* as a direct consequence that a single diffusion tensor does not properly represent the dMRI signal (even when a single fixel is present) for b-values higher than 1ms/μm^2^, needing more tensors to fit the per voxel signal (Karaman et al., 2023).

Between the MTM-based methods, MRDS offers a balanced trade-off in terms of model complexity and accuracy when using short-acquisition-time clinical multi-shell dMRI data (Coronado-Leija et al., 2017). MRDS has proven to be a noise-robust and accurate multi-fixel method for estimating the directions of the fixels and their metrics. In addition, MRDS has been histologically validated in a rat model of unilateral retinal ischemia in which only one of the optic nerves was damaged. This nerve lesion was correctly detected by MRDS at the region where the optic nerves cross (optic chiasm) (Rojas-Vite et al., 2019). Moreover, MRDS has shown to be capable of recognizing 3 fiber populations in regions-of-interest (ROI) like the centrum semioval (Hernandez-Gutierrez et al., 2021) when using clinical *in vivo* multi-shell dMRI data. A recent work (Karaman et al., 2023) has proposed to use the **T**rack **O**rientation **D**ensity **I**maging (TODI) (Dhollander et al., 2014) as a useful spatial regularizer for a more accurate and robust estimation of *N* in MRDS. The **T**rack **O**rientation **D**istribution (TOD) image estimated with TODI presents an increased amount of spatial consistency compared with the fiber orientation distribution (FOD) image obtained with constrained spherical deconvolution (CSD) (Dhollander et al., 2014).

In this paper, we propose a novel tractometry pipeline to address several current limitations of tractometry informed with multi-fixed methods. Our proposed pipeline combines multi-tensor fixel-based metrics estimated with MRDS and the Tractoflow (Theaud et al., 2020) and Tractometry_flow (Cousineau et al., 2017; Kurtzer et al., 2017; Di Tommaso et al., 2017) pipelines. The proposed pipeline provides fixel-based tensor metrics that are robust to crossing fibers and noise. Provided fixel-based metrics have the potential to be biomarkers for pathologies like demyelination and can be useful for the characterization and study of underlying WM anomalies in patients with pathologies such as MS. Most of the previous tractometry studies in pathology used DTI metrics, then, our multi-tensor pipeline results can be straightforwardly situated in their context and compared with them. Finally, the pipeline is tested on both synthetic phantom dMRI data and clinical dMRI *in vivo* data from a large healthy control and MS groups with a scan-rescan experiment, highlighting the robustness and potential of our approach when studying WM anomalies in patients with such neurological disorders.

## 2 Methods

In this section, we describe the simulation of the synthetic phantom and the acquired *in-vivo* dMRI data. We also explain each step in the proposed pipeline.

### 2.1 Synthetic data

**Figure 3:**
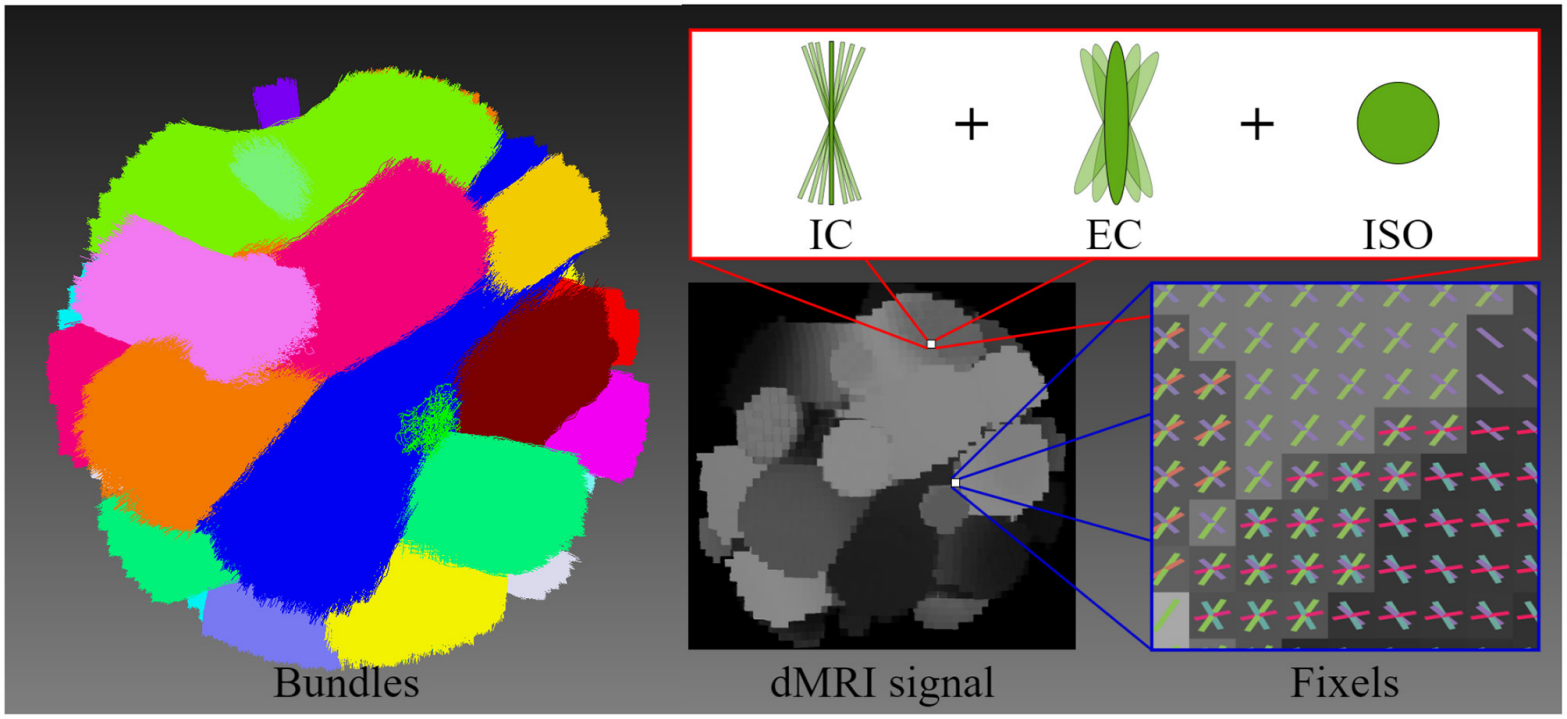
This figure illustrates the geometry of the diffusion MRI (dMRI) phantom, the multi-compartment model employed for signal generation, and the resulting signal. The geometric structure of the phantom is composed of 20 bundles, highlighting each bundle with a different color. The multi-compartment model (intracellular, extracellular, and free water compartments) that is used to generate the shown dMRI signal at each voxel includes axonal dispersion. The figure illustrates the orientations of fixels, depicting the crossing fiber configuration and distribution.

A synthetic phantom was generated based on the geometry of a previously published dMRI phantom (Caruyer et al., 2014), see Figure 3. The size of the phantom is 50 × 50 × 50 voxels with an isotropic dimension of 1.0mm. Similar to Caruyer et al. (2014), our synthetic phantom has 20 distinct bundles showing a complex fiber crossing configuration and volume contamination with **C**erebro**S**pinal **F**luid (CSF). Each bundle in the phantom exhibits unique diffusivities and axonal dispersion characteristics. The diffusivities of each bundle were tuned to mimic those found in healthy human brains (Coelho et al., 2022).

We have simulated a phantom dMRI signal for each individual bundle and the whole volume signal without noise and without dispersion. Then, DTI was fitted to each individual bundle signal as well as the whole dMRI signal, and the tensor metrics were extracted. This simulated dataset was employed as *Gold Standard* (GS) to compare results with the experiments on *in-vivo* dMRI data. A multi-compartment model also known as Standard Model (SM) (Ferizi et al., 2016; Novikov et al., 2019), was adopted to simulate this phantom signal by including three types of microstructural environments: intracellular (IC), extracellular (EC), and isotropic (ISO). Each environment was simulated with a given volume fraction denoted by *f*_ic_, *f*_ec_ and *f*_iso_, respectively. The IC space was modeled with cylinders of zero radius (*sticks*), the EC space with a cylindrically symmetric tensor (*zeppelin*), and finally, the ISO space was modeled as a free diffusion compartment (*ball*) (Panagiotaki et al., 2012; Ferizi et al., 2013a,b).

Three datasets were generated with known GS. The radial EC diffusivities were simulated based on Fieremans et al. (2012). Thus, the EC space tortuosity $D_{0}/D_{\text{ec}}^{⊥}$, which quantifies how the diffusion is affected by cellular and extracellular structures within tissue, was defined as the ratio of free diffusivity $D_{0}=2\mu {\text{m}}^{2}/\text{ms}$ over the EC diffusivity $D_{\text{ec}}^{⊥}$. Therefore, the intracellular volume fraction *f*_ic_ was most sensitive to axonal loss. Besides, it was most sensitive to demyelination. The first dataset incorporated $D_{\text{ic}}^{∥}$ and $D_{\text{ec}}^{∥}$ diffusivities within a healthy range sampling a Gaussian distribution with a mean of 2μm^2^/ms and variance of 0.01μm^2^/ms, while $D_{\text{ec}}^{⊥}=0.48\mu {\text{m}}^{2}/\text{ms}$, *f*_ic_ = 0.65 and *f*_ec_ = 1−*f*_ic_. On the other hand, the second dataset simulated, in some bundles, conditions associated with demyelination on MS. Specifically, in regions with demyelination *f*_ic_ = 0.55 and $D_{\text{ec}}^{⊥}=0.71\mu {\text{m}}^{2}/\text{ms}$, while in regions without damage, the values remained the same as in the first dataset. Finally, our third dataset simulated conditions related to axonal loss. For this case, *f*_ic_ = 0.35 and $D_{\text{ec}}^{⊥}=0.59\mu {\text{m}}^{2}/\text{ms}$ in regions with lesion and regions without lesion maintained the same control values as the first dataset. All datasets were generated with a high and realistic noise level (*SNR* = 12). The isotropic diffusivity *D*_iso_ and volume fraction *f*_iso_ were fixed equal to 3μm^2^/ms and 0.05, respectively. Axonal dispersion was modeled with a Watson distribution (Jespersen et al., 2012, 2018). The κ value of each bundle used as the parameter for the Watson distribution was sampled from a Gaussian distribution with mean 20 and variance 0.01. Lastly, we used the same protocol of the *in-vivo* data described below.

The 13th bundle of the phantom was selected to compare the three scenarios above because bundle 13 crosses with 2 and 3 bundles at different places. In the datasets simulating damages, the lesion was simulated in a spot of the bundle represented by the red region, while the diffusivities outside the lesion remained the same as in the control case.

### 2.2 *In-vivo* data

Two groups of participants were recruited from the University of Sherbrooke (UdS) and the Center Hospitalier Universitaire of Sherbrooke (CHUS) community. The first group was a healthy control (HC) group with 26 adults, and the second group has 22 relapsing-remitting MS patients. Both groups had a gender proportion of 75% women and 25% men. Diffusion MRI data was acquired using a clinical 3T MRI scanner (Ingenia, Philips Healthcare) using a 32-channel head coil. Each subject was scanned 5 times over 6 months and a 4-week interval (±1 week) with a total acquisition time of 20 minutes for each session. MRI acquisitions were obtained for each subject at roughly the same time daily to mitigate potential diurnal impacts, i.e. morning subjects underwent all sessions in the morning with a permissible 2-3-hour variation. Finally, 6 of the 26 healthy control subjects were discarded for several reasons, including problems during the scan or processing. Thus, the HC group employed for the experiments had 20 subjects.

All MRI images were aligned respect to the anterior commissure-posterior commissure plane (AC-PC), which is an anatomical reference defined by two small bundles in the brain. One bundle located in the front part of the brain, and the other in the back. This ensured consistency in the orientation and position of the images when analyzing them across scans and subjects. In addition, 3 type of data were included:

- Anatomical 3D T1-weighted image (T1): an MPRAGE image was acquired axially at 1.0mm isotropic resolution.
- Multi-shell diffusion-weighted images (DWI): these images were acquired with a single-shot EPI spin-echo sequence at 2.0mm isotropic resolution. The acquisition scheme included 100 unique gradient directions uniformly distributed over and across 3 shells at b-values *b* = 0.3ms/μm^2^ (8 directions), *b* = 1ms/μm^2^ (32 directions) and *b* = 2ms/μm^2^ (60 directions) with 7 non-diffusion-weighted images (*b* = 0) for a total of 107 diffusion volumes. An additional reversed phase-encoded *b* = 0 image was acquired after the DWI acquisition with the same geometry to correct EPI distortions.
- Inhomogeneous magnetization transfer images (ihMT): these images were acquired using a 3D segmented-EPI gradient-echo sequence with different magnetization transfer (MT) preparation pulses. They have 2 × 2 mm resolution and 65 slices with 2.0 mm thickness.

Finally, all images have been subjected to visual quality assessment. A detailed and more extensive data description can be found in Edde et al. (2023).

### 2.3 Pipeline

**Figure 4:**
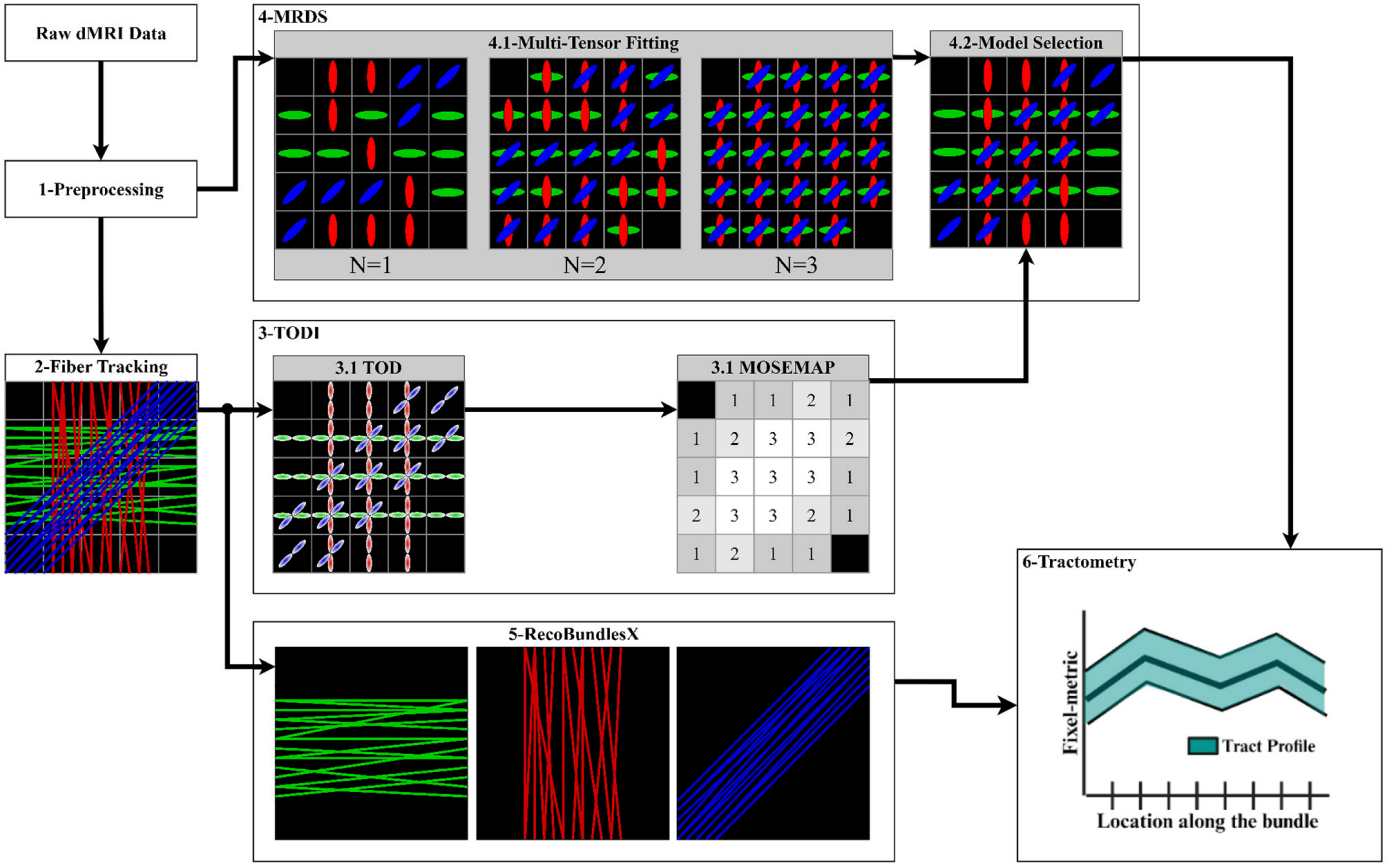
Proposed pipeline to extract the tract profiles using the MRDS fixel-based multi-tensor metrics. (1) Input data is denoised, aligned, corrected, normalized, cropped, and resampled. Brain, WM, and lesion masks are extracted. (2) The CSD method is fitted to obtain an FOD image, and a local tracking technique is used to estimate the streamlines. The WM mask is used to seed the streamlines. (3) TODI maps the tractogram into a NuFO map, which MRDS exploits as a model selection map in the MTM fitting. (4.1) MTM is fitted to the dMRI data using MRDS. MTM parameters are estimated for *N* = 1, *N* = 2, and *N* = 3, resulting in 3 MTFs. (4.2) The TODI NuFO map is used on the estimated MTFs to choose a value of *N* that better describes the diffusion signal at each voxel. TODI helps to refine the MTF with the spatial information provided by the tractogram (Karaman et al., 2023). Multi-tensor fixel-based metrics are computed from the MTF derived from the model selection. (5) The tractogram is segmented into major fiber bundles using the *RecoBundlesX* pipeline. (6) Segmented bundles and estimated fixel-metrics are the input of the *Tractometry_flow* pipeline to assess a tract profile for every combination of the bundles and metrics.

The data processing pipeline consists of 6 key steps described in sequential order in the following subsections, see Figure 4:

#### 2.3.1 Preprocessing

The preprocessing of the dMRI data was performed using the *Tractoflow* pipeline (Theaud et al., 2020). This includes the brain and WM masks extraction, T1 registration and tractography.

The dMRI data was denoised using the MP-PCA (Veraart et al., 2016) method. Brain deformation induced by magnetic field susceptibility artifacts was corrected (Andersson et al., 2003). Motion artifacts corrections and slice-wise outlier detection were performed (Andersson and Sotiropoulos, 2016). Image intensities were normalized to reduce the bias by the magnetic field (Tustison et al., 2010). The brain mask was obtained from the bet command from FSL (Smith, 2002). Specifically, Tractoflow performed an extraction on the b = 0 image. Then, the obtained mask was applied to the whole DWI to remove the skull and prepare the DWI for the T1 Registration. Tractoflow performed brain extraction after Eddy/Topup correction to have a distortion-free brain mask.

Tractoflow processed the T1 image using eight different steps. First, Tractoflow preprocessed the T1 image including denoising, correcting and resampling steps for the T1 image. Then, the T1 image was registered on the b = 0 and FA images using the nonlinear SyN ANTs (antsRegistration) multivariate option, where the T1 image is set as moving image and the b0 and FA images are set as target images. After registration, Tractoflow extracted gray matter (GM), white matter (WM) and cerebrospinal fluid (CSF) partial volume masks using fast from FSL. These maps were used to compute the exclusion and inclusion maps for tractography (Girard et al., 2014), which are anatomical constraints for the tracking (Smith et al., 2012; Girard et al., 2014).

#### 2.3.2 Fiber tracking

The fiber tracking was also done using the *Tractoflow* pipeline. The seeding mask employed in the tractography was the obtained WM mask. The tractogram was generated employing the anatomically constrained particle filter tracking (PFT) algorithm (Girard et al., 2014). This algorithm utilized the FOD image obtained with the Multi-Shell Multi-Tissue Constrained Spherical Deconvolution (MSMT-CSD) (Jeurissen et al., 2014), along with the inclusion map, exclusion map, and a seeding mask to guide the tractography process. The seeding mask employed in the tractography was the extracted WM mask. A fully detailed explanation of the whole Tractoflow pipeline can be found in Theaud et al. (2020).

Additionally, *Tractoflow* includes strategies to avoid premature track termination when tracking MS patients. The seeding mask in MS patients was filled using a lesion-corrected WM mask. During the tracking process, if a peak in the FOD image is coherent and well-defined, the tracking continues even if the voxel is inside a WM lesion, increasing anatomical accuracy and consistency in obtained tractograms for MS patients. This step can be omitted as the tractogram can be generated with any fiber tracking technique.

#### 2.3.3 TODI as model selector

The tractogram from fiber tracking was then processed with the TODI method to obtain a TOD image. Subsequently, the resulting TOD image was segmented to produce discrete fixels (Smith et al., 2013). Then, the fixel-based image was converted into a **Nu**mber of **F**iber **O**rientations (NuFO) scalar image, where the number of fixels was counted in each voxel. A threshold peak amplitude was utilized to prune the spurious peaks, such that any lobe for which the maximal peak amplitude was smaller than 0.1 was omitted. Finally, this NuFO image was used as input MOSEMAP in MRDS, which is better described in the next step (Section 2.3.4).

#### 2.3.4 Multi-tensor Fitting

The MTM represents the diffusion signal *S*_*i*_ at each voxel as:


(1) $$\begin{array}{l} S_{i}/S_{0}=\overset{N}{\underset{j=1}{\sum }}\alpha_{j}exp\left(-b_{i}g_{i}^{T}D_{j}g_{i}\right),i=1,\ldots M, \end{array}$$

where *M* is the number of unitary gradient orientations ***g***_*i*_, *N* is the number of tensors, and α_*j*_ is the fraction of the *j*-th diffusion tensor ***D***_*j*_. Assuming axial symmetry, then ***D***_*j*_ is parameterized by the unitary principal diffusion direction (PDD) **θ_*j*_**, the axial ($\lambda_{j}^{∥}$) and radial ($\lambda_{j}^{⊥}$) diffusivities, such that:


(2) $$\begin{array}{l} g_{i}^{T}D_{j}g_{i}=\lambda_{j}^{∥}{\left(\theta_{j}\cdot g_{i}\right)}^{2}+\lambda_{j}^{⊥}\left[1-{\left(\theta_{j}\cdot g_{i}\right)}^{2}\right] \end{array}$$

The bundle-specific parameters of the MTM were non-linearly estimated using the MRDS (Coronado-Leija et al., 2017) method for *N* = 1, *N* = 2, and *N* = 3, resulting in 3 multi-tensor fields (MTFs), see Figure 4. More than three fixels can be estimated, albeit with increased computation time and reduced precision for the estimated parameters. Besides, *N* ≤ 3 has been reported to be a reasonable threshold (Jeurissen et al., 2013). Initial diffusivities for the non-linear estimation of parameters in Equation 2 were obtained from DTI at brain WM voxels with a high probability of containing only one fiber.

Given that high b-value diffusion signals are not fully represented with the diffusion tensor, causing an overestimation in *N*. Thus, the original statistical model selection in MRDS, which provides a model selection map (MOSEMAP) with the value of *N* that better describes the diffusion signal at each voxel, is replaced for the NuFO scalar map obtained with TODI in step 2.3.3. The TODI NuFO scalar map merges the 3 MTFs into a reevaluated and refined MTF with the spatially smoothed information provided by the tractogram (Karaman et al., 2023), see Figure 4. From this improved MTF, fixel-FA, fixel-MD, fixel-AD, and fixel-RD maps were generated. The fixel-FA map maintains the same spatial dimensions as the original DWI Each voxel may contain multiple tensors. Then, an extra layer was added to store the multiple fixel-FA values obtained at each voxel. The scalar fixel-FA value was obtained for every tensor within a voxel, computed as the standard FA (Basser et al., 1994). This resulted in a 4D-dimensional fixel-FA map. The computation of fixel-RD, fixel-AD and fixel-MD maps was analogous to the computation of the fixel-FA map. Similarly, a map storing the PPDs of the MTF was computed. These maps were used as input for the tractometry step.

#### 2.3.5 Tractogram segmentation

The tractogram was segmented into major bundles employing the RecoBundlesX (St-Onge et al., 2023, 2020; Kurtzer et al., 2017; Di Tommaso et al., 2017) pipeline, see Figure 4. RecoBundlesX recognizes bundles by comparing the subject's tractogram with a template (or atlas) through a similarity metric based on their shapes. This algorithm is re-evaluated multiple times with parameter variations and label fusion because RecoBundlesX is a multi-atlas and multi-parameter approach. We used the atlas in Rheault (2023), which is designed specifically to be used with RecoBundlesX, and it was built from delineation informed with anatomical priors (Catani and Thiebaut de Schotten, 2008). After RecoBundlesX identified a large number of WM bundles, tracks were visually inspected to ensure their quality. The Superior Longitudinal Fasciculus (SLF), Arcuate Fasciculus (AF), Pyramidal Tract (PYT), Inferior Longitudinal Fasciculus (ILF), Inferior Fronto-Occipital Fasciculus (IFOF), Middle Cerebellar Peduncle (MCP) and Cingulum (CG) bundles were selected to showcase the pipeline's capabilities. Selected bundles comprise a large set covering most of the brain, showing complex crossing fiber configurations, which is why they are frequently studied in the literature (Yeatman et al., 2012; Mishra et al., 2014; Chamberland et al., 2019; Winter et al., 2021).

In the experiments with MS patients, we have chosen the AF, ILF, IFOF and PYT bundles, which have clinical implications in the context of MS studies (Filippi and Rocca, 2011). The AF bundle connects the frontal and temporal lobes, crucial in speech communication. On the other hand, the ILF bundle connects the occipital and temporal lobes. Its functionality includes visual processing, tracking and recognition of objects and obstacles. Like AF and ILF, the IFOF bundle is involved in speech communication and visual processing tasks, transporting signals from the frontal to occipital and temporal lobes. The PYT connects the spinal cord with the cerebral cortex. It is essential in voluntary control movements. Therefore, when MS lesions appear in the AF, ILF, IFOF, and PYT bundles, several symptoms are experienced by MS patients. These symptoms include difficulties in speech and comprehension, visual deterioration, visual memory problems, attention issues, and affected motor coordination.

#### 2.3.6 Tractometry with fixel-based metrics

The proposed pipeline employed the *Tractometry_flow* (Cousineau et al., 2017; Kurtzer et al., 2017; Di Tommaso et al., 2017) pipeline, which delivers metric maps along each individual input bundle. Then, each metric map was projected through every bundle to obtain a tract profile. We adapted the *Tractometry_flow* pipeline to support multi-tensor fixel-based metrics. The *closest-fixel-only* (Raffelt et al., 2015) strategy was used to map the contribution of the multi-fixels estimated by MRDS to a given streamline.

### 2.4 Experiments

We designed three experiments to study the behavior of the pipeline:

- *Experiment I:* our proposed pipeline is tested on the three synthetic phantom datasets described before, simulating healthy tissue, demyelination and axonal degeneration. Estimated multi-tensor fixel-based metrics and tractometry results are compared with the known GS of the phantom. Relative errors obtained using the formula $error=\frac{\left|value_{real}-value_{estimated}\right|}{value_{real}}$ are reported in the results.
- *Experiment II:* the pipeline is used to study the robustness to crossing fibers of the tract profiles in the *in-vivo* healthy control group. Obtained tract profiles informed with multi-tensor fixel-based metrics of the 100 total scans (20 subjects scanned 5 times each one) are group-averaged and compared to DTI-derived tract profiles.
- *Experiment III:* the pipeline is employed to study variations of metrics across the AF, ILF, IFOF and PYT, which are relevant bundles in the study of relapsing-remitting MS. As the location and severity of the MS lesions are different for every individual patient, it is irrelevant to make a group-averaged study of the MS group of patients. Instead, we have manually selected patients 004 and 022 because they have the most severe and larger white matter coverage of lesions present in the WM. These particular patients are chosen in an effort to maximize the difference between the healthy control group and the MS patient tract profiles. Individual patient tract profiles informed with multi-tensor fixel-based metrics are compared with the group-averaged tract profiles in order to exhibit differences.

## 3 Results

### 3.1 Experiments on synthetic data

**Figure 5:**
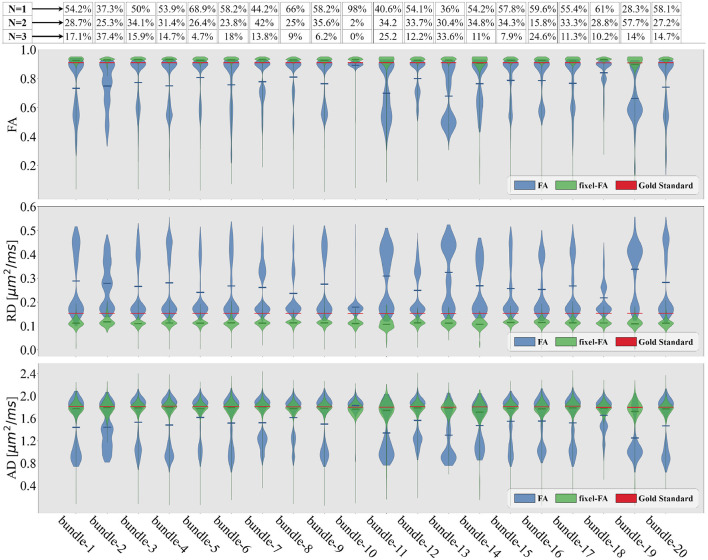
Violin plots of the estimated tensor and multi-tensor fixel-based metric maps along each bundle of the control synthetic dataset (control values without lesions). Tensor metrics are estimated with DTI (blue violin) and multi-tensor fixel-based metrics with MRDS (green violins). Obtained metrics are compared with the GS (red line). The bundle's average crossing fibers composition in the GS is indicated in the proportions containing *N* = 1 fiber, *N* = 2, or *N* = 3 fibers at the top of the figure.

In Figure 5, we show violin plots comparing single-tensor (blue) and multi-tensor (green) metrics. Horizontal lines refer to the GS (red) and the mean of each distribution. Single-tensor metrics exhibit several discrepancies with respect to the GS, most DTI distributions are bimodal, such that one of the peaks is close to the GS, while the other is underestimated for FA and AD, and overestimated for RD.

For each bundle, we accounted for the proportion of voxels containing 1, 2, and 3 fiber populations using the NuFO map obtained with TODI, i.e., we accounted for the proportions of *N*. These proportions are at the top of Figure 5. By inspecting percentages of *N* shown in Figure 5, it is reasonable to assume that DTI bimodality is caused by crossing fiber biases. In Figure 5, bundles with a high proportion of *N* = 2 and *N* = 3 have a more pronounced bimodality; this is particularly evident for the 13th bundle. In contrast, it can be seen in Figure 5 that the mean of the estimated fixel-FA and fixel-AD are similar to the GS value in all bundles. The relative error of fixel-FA and fixel-AD is around 10% as it is reported in Table 1, reaching a relative error as low as 2.7% in some bundles where the average relative error is 5.6%. It is important to note that, for fixel-based metrics, the relative error of bundles with a high count of 2 and 3 crossing fibers is similar to the relative error in bundles exposing mostly single fiber composition. As an example, percentages shown in Figure 5 exhibit that bundles 5 and 10 mainly have no crossing fibers, while bundle 11 has, for the most part, crossing fibers. However, the relative error of fixel-FA for bundles 5, 10, and 11 in Table 1 are around 3%. Even for the bundle 13, which is one of the most challenging bundle as it has a high proportion of crossing fibers, the relative error remains at 7%. Values in Table 1 exhibit a higher relative error for fixel-RD compared with fixel-AD and fixel-FA, but still less relative error than RD in general. Additionally, bundle 2 shows abnormal relative errors compared to the other bundles.

**Table 1:** Relative errors of the violin plots for the tensor (blue) and multi-tensor (green) metric maps estimated with DTI and MRDS, respectively.

**Metric**	**FA**	**Fixel-FA**	**RD**	**Fixel-RD**	**AD**	**Fixel-AD**
**Label**						
1	19.4%	2.7%	89.0%	26.7%	23.9%	4.2%
2	17.6%	31.5%	82.0%	46.9%	22.5%	33.2%
3	15.1%	3.4%	74.1%	28.4%	20.2%	4.2%
4	17.6%	3.0%	83.9%	26.7%	22.8%	4.2%
5	11.3%	3.2%	58.1%	26.8%	16.0%	3.9%
6	16.7%	4.3%	75.1%	27.9%	20.6%	5.9%
7	14.5%	3.9%	71.1%	27.5%	20.1%	4.7%
8	10.9%	2.7%	54.9%	25.8%	15.1%	3.8%
9	15.9%	2.7%	80.3%	26.0%	21.6%	3.4%
10	2.2%	2.7%	18.0%	27.5%	5.3%	4.4%
11	23.1%	3.3%	102.9%	29.0%	27.7%	5.6%
12	12.1%	3.7%	63.0%	26.1%	17.7%	4.7%
13	25.4%	7.5%	112.5%	30.0%	29.7%	9.1%
14	15.8%	2.8%	75.9%	28.2%	20.3%	5.1%
15	13.4%	4.5%	68.1%	25.9%	18.5%	5.5%
16	13.6%	7.4%	66.6%	28.3%	18.5%	9.2%
17	15.8%	3.7%	75.0%	26.1%	20.5%	5.1%
18	7.6%	10.2%	43.2%	32.1%	12.2%	11.3%
19	27.0%	4.9%	121.8%	27.8%	32.0%	6.5%
20	18.6%	5.3%	85.5%	29.2%	22.7%	6.8%
Mean	15.6%	5.6%	75.0%	28.6%	20.3%	7.0%

Looking at the obtained tractogram, the streamline count for bundle number 2 after segmentation is 104, which is insufficient to cover the whole bundle's volume, resulting in an increased relative error. Thus, results in bundle 2 should be interpreted with caution because of the low number of streamlines. Bundle 10 has almost 100% single fiber composition. It is the only bundle where the relative error of RD is less than the one reported in fixel-RD. This suggests that, in the absence of crossing fibers, DTI's RD may be more accurate than fixel-RD. Besides, fixel-RD violin plots in Figure 5 indicate that, in general, fixel-RD tends to underestimate the GS value, which is congruent with the relative errors reported in Table 1. In the MTM fitting with MRDS the isotropic volume fraction is overestimated, see Appendix A. Since the synthetic data was generated using a multi-compartment model and MTM does not fully represent the signal for the b-values in our protocol, then the ISO compartment may be partially explaining the contribution of the EC compartment (see Appendix A for more details). Therefore, this underestimation of the fixel-RD metric might be related to the overestimation of the isotropic volume fraction.

**Figure 6:**
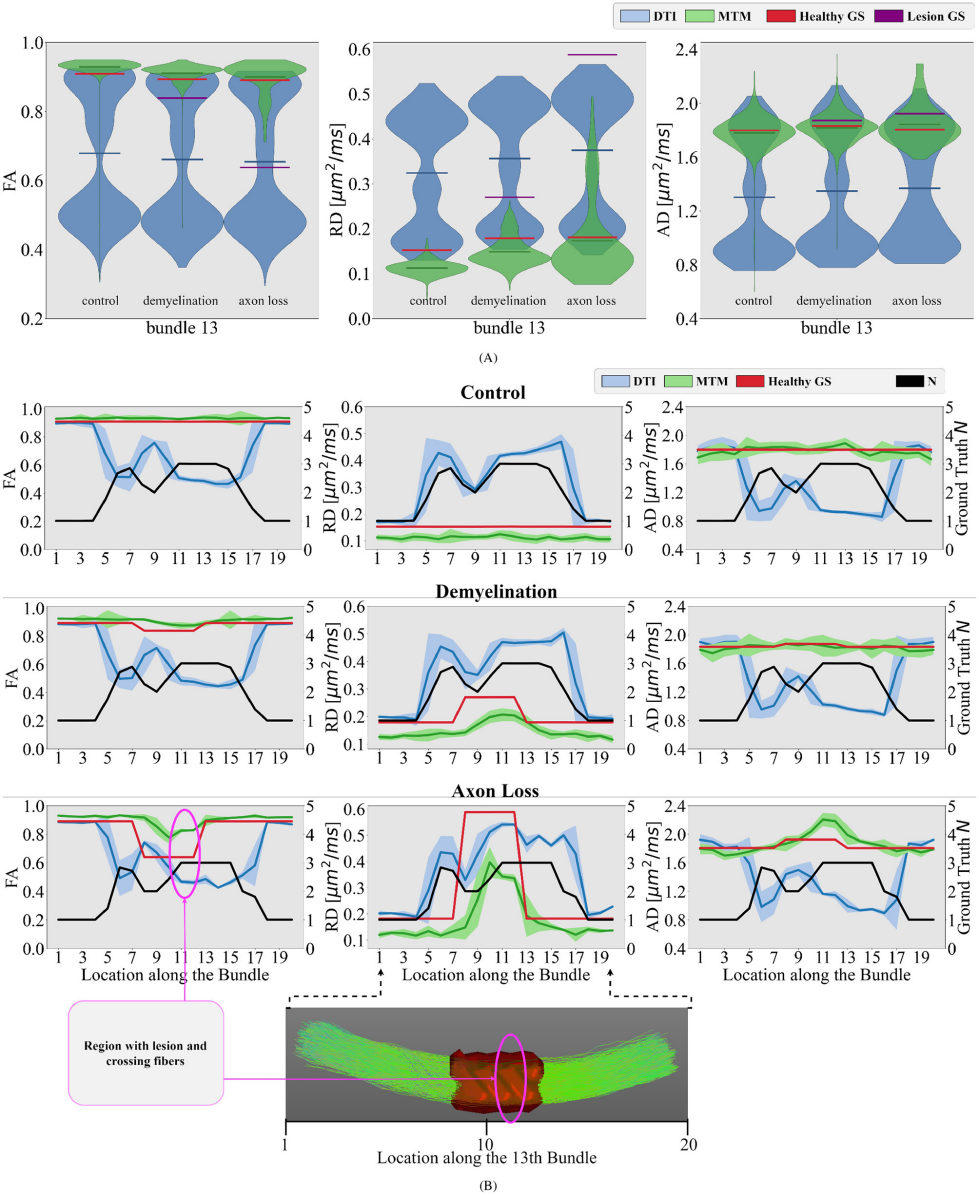
Differences between different simulated scenarios are studied in the bundle number 13 of the phantom. **(A)** Violin plots of the estimated single-tensor (blue) and multi-tensor (green) fixel-based metrics for each scenario. The mean of the distributions is represented as a horizontal line matching the color of the distribution. The GS of the healthy tissue and damaged tissue are represented as red and purple horizontal lines, respectively. **(B)** Tract profiles resulting from projecting the estimated single- and multi-tensor fixel-based metrics along the 13th bundle. Every row represents a different scenario (control, demyelination, and axon loss), while every row represents a different measure (FA, RD, and AD). The GS value of *N* (black curve) and tensor metrics (red curve) are also projected on the bundle number 13, showing the actual underlying fiber configuration along this bundle for comparison. A region with lesions and crossing fibers is highlighted in pink.

Similar to Figure 5, in Figure 6 violin plots on the 13th bundle are reported for the 3 simulated scenarios detailed in Section 2.1: healthy control case, demyelination, and axonal loss. Additionally, tractometry results on the same bundle for the 3 different scenarios can be found in Figure 6B. In the healthy control scenario, the limitations of DTI in capturing the overall WM microstructure configuration are evident. Tract profiles informed with standard DTI metrics are biased by crossing fibers as FA, RD, and AD tract profiles have variations along the bundle, while the GS does not. In particular, FA tract profile decreases and RD tract profile increases when the value of *N* increases, see Figure 6B. In contrast, the robustness of the multi-tensor fixel-based metrics estimated with MRDS is evident as they provide tract profiles independent of the underlying fiber configuration, see Figure 6B.

In the demyelination scenario, results with DTI metrics in Figure 6 showed limited sensitivity to changes in the WM microstructure. In the region with a lesion, tract profiles exhibit variations, but they do not correspond with the GS. Contrarily, results with multi-tensor fixel-based metrics show enhanced sensitivity, detecting reductions in FA and increase in RD associated with simulated demyelination while maintaining robustness to noise and crossing fibers, see Figure 6. Like the demyelination scenario, DTI metrics exhibit limitations in detecting axonal loss, particularly in regions with crossing fibers. Despite the differences in the three simulated scenarios, results in Figure 6 show no substantial differences in DTI metrics. This makes it impossible to distinguish between different scenarios. Results with multi-tensor fixel-based metrics are less contaminated by fiber crossing artifacts, which allows to detect variations in the tract profiles related to lesions. Obtained tract profiles informed with fixel-based metrics underestimate the GS RD, which is expected and congruent with the results investigated in Figure 5. Although results with multi-tensor fixel-based metrics overestimate the GS FA and underestimate the GS RD, they are accurate in shape and sensitive to small variations.

### 3.2 Experiments on *in-vivo* data

**Figure 7:**
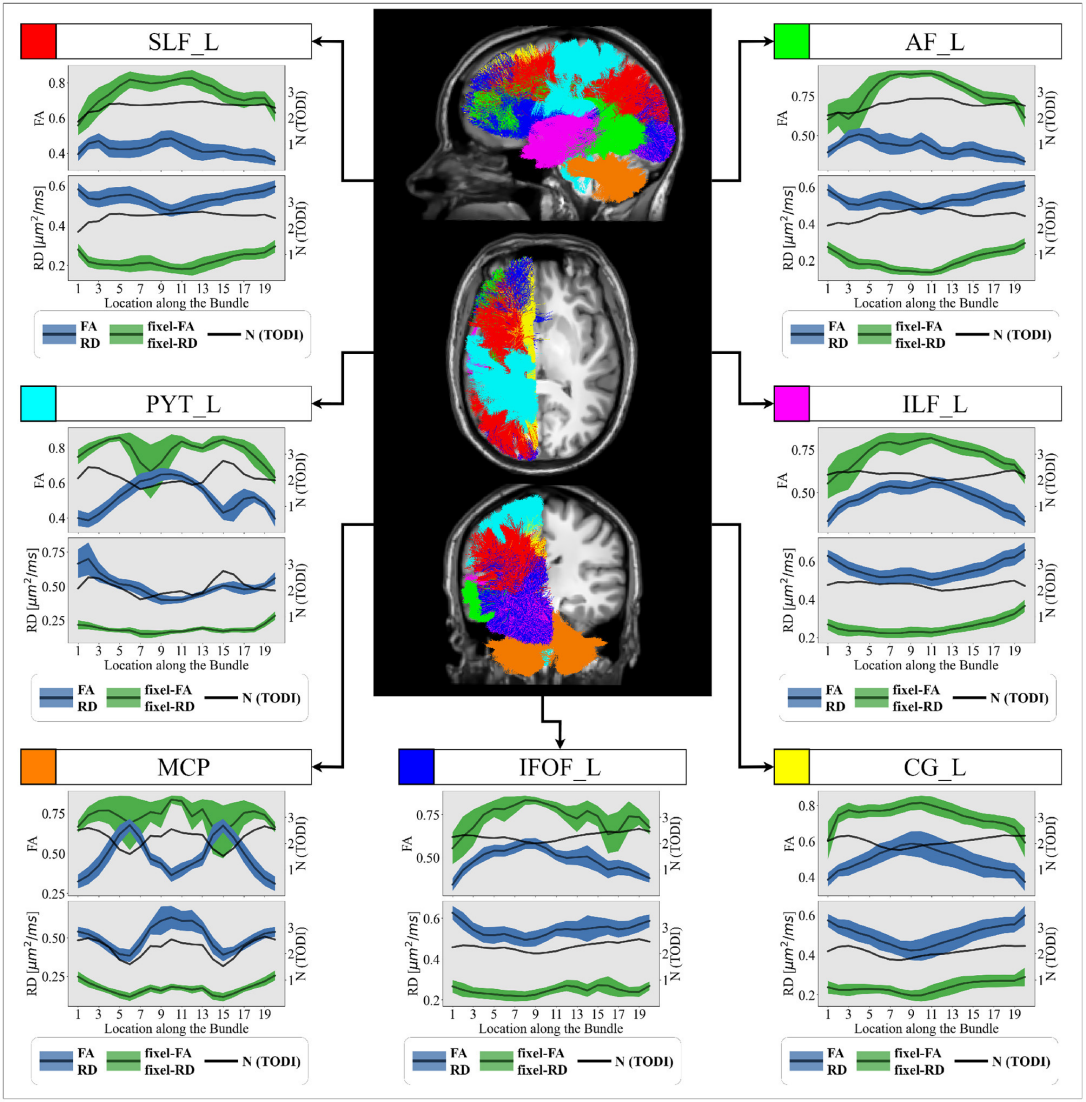
Group-averaged tract profiles of the 100 healthy control scans (comprising 20 participants scanned five times over 6 months) along several segmented WM bundles: the (red) SLF_L, (cyan) PYT_L, (orange) MCP, (blue) IFOF_L, (yellow) CG_L (magenta) ILF_L and (green) AF_L bundles. The mean and variance of the tract profiles obtained with DTI-derived metrics (green) and MTM-derived fixel-based metrics (blue) are represented with a bold line and a shaded area, respectively. The estimated number of crossing fibers (*N*) using TODI along each selected bundle is represented by an black curve. The axial, coronal and sagittal views a control subject's T1 image are provided as anatomical references.

For experiments on *in-vivo* data, we focus only on FA and RD metrics and their fixel-based counterparts, as MS research and literature report that FA and RD are potential biomarkers closely related to microstructure anomalies and demyelination (Song et al., 2002, 2005). Figure 7 illustrates tract profiles for different major bundles in the left hemisphere of the healthy participants.

According to Table 2, tract profiles obtained with MRDS fixel-FA and fixel-RD metrics show an overall reduction in the correlation with the value of *N* compared to FA and RD metrics. Tract FA profile decreases in locations where *N* is high and vice versa. In contrast, tract fixel-FA profiles exhibit more robustness to crossing fibers. Additionally, tract profiles informed with fixel-based multi-tensor metrics show FA similar to the ones reported in healthy WM of human brain. Based on the literature, FA values in the healthy human brain WM generally range between 0.60 and 0.85, depending on the specific tract or region. For example, FA in the corpus callosum was reported to be between 0.72 and 0.78 (Westlye et al., 2010), and between 0.73 and 0.76. FA in the internal capsule was reported to be between 0.70 and 0.80 (Mukherjee et al., 2008), and around 0.75. Finally, FA in frontal WM was found to be between 0.60 and 0.70 (Westlye et al., 2010), and between 0.60 and 0.65. Results on the healthy control group dataset follow the patterns observed in the experiments on the control synthetic dataset. Like Figure 5, tract profiles obtained with DTI-based metrics consistently show lower FA and higher RD values compared to the fixel-based metrics across every bundle.

**Table 2:** Pearson correlation coefficients between the tract profiles of MRDS fixel-based metrics (green curves), DTI metrics (blue curves) and the NuFO value of *N* (black curve) in Figure 7. Each metric was evaluated across the same set of bundles as in Figure 7.

**Correlation with *N***	**SLF_L**	**AF_L**	**PYT_L**	**ILF_L**	**MCP**	**IFOF_L**	**CG_L**
FA	0.0967	-0.2601	-0.7193	-0.4657	-0.9682	-0.7553	-0.8593
Fixel-FA	0.8311	0.8631	0.5264	-0.4285	0.5364	-0.5641	-0.6131
RD	-0.4528	-0.2934	0.4773	0.3926	0.7444	0.5921	0.8717
Fixel-RD	-0.4994	-0.5395	0.1480	0.1433	0.8568	0.4370	0.7228

Figure 7 shows average tract profiles computed from the HC cohort, which includes different subjects scanned in different time stamps. Tract profiles in Figure 7 show visually low variability overall. In Table C1, the standard deviation (SD) is presented for tract profiles informed with both fixel-based and DTI metrics. The SDs are computed within-subject and between-subject for each bundle and each section of the bundle. The SD from tract fixel-FA profiles is generally higher than the tract FA profiles, though they remain comparable overall. Additionally, Table C2 presents the results of the ANOVA test conducted to compare the mean of 5 tract fixel-FA and fixel-RD profiles resulting from the five scans of sub-015 (one of the subjects exhibiting the highest variability), see Appendix C. The ANOVA test shows the F-statistic and p-value across the 20 locations (labels) of the selected bundles. The results revealed significant differences in the means for various bundles at specific labels. Notably, Label 2 exhibited a statistically significant effect in the PYT_L bundle with an F-statistic of 3.9618 (p = 3.84E-04) and in the ILF_L bundle (F-statistic = 1.6415, *p* = 1.69E-02). Similarly, Label 8 demonstrated significant findings in the SLF_L (F-statistic = 4.6286, *p* = 2.74E-04) and MCP (F-statistic = 15.2726, *p* = 2.82E-06) bundles. Additionally, Label 12 showed highly significant results in the AF_L (F-statistic = 12.99599, *p* = 5.45E-07) and PYT_L (F-statistic = 10.1636, *p* = 1.48E-07) bundles.

### 3.3 Experiments on relapsing-remitting MS data

**Figure 8:**
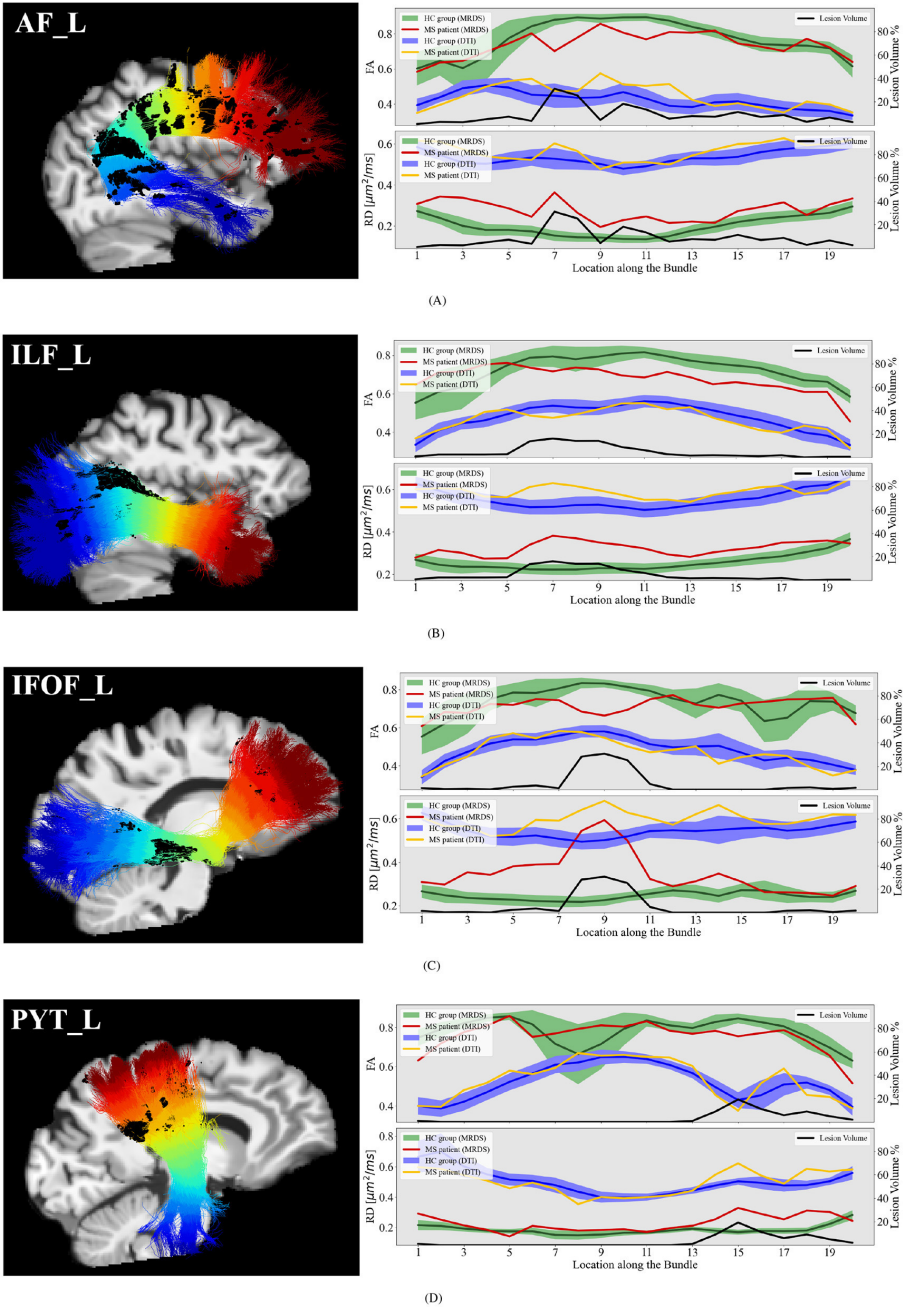
Single MS patient fixel-FA and fixel-RD tract profiles (red lines) compared with the group-averaged tract profiles (green lines with shaded areas) of the 100 healthy control subjects (comprising 20 participants scanned 5 times) along several segmented WM bundles: the **(A)** AF_L, **(B)** ILF_L, **(C)** IFOF_L, and **(D)** PYT_L. Tract profiles of lesion volume (black lines) are reported to exhibit the potential relationship between the fluctuations in the tract profiles and the lesion. The sagittal view of the MS patient's T1 image, the bundle, and the lesions (dark blue surfaces) are provided as anatomical references.

Our pipeline was applied to the MS dataset for a set of relevant bundles in the context of MS studies: AF, ILF, IFOF, and PYT. Differences between MS patients and HC group-averaged tract profiles are studied in Figure 8. In locations adjacent to lesions, fixel-FA tract profiles show lower values than the healthy control group. Moreover, on the ILF and IFOF bundles, fixel-FA values are beyond the second variance, which may indicate degradation of the WM integrity. Besides, fixel-RD tract profiles are consistently elevated compared to healthy controls in regions with lesions, suggesting widespread expected demyelination. The spatial extent of the lesions correlates with the extent of the changes in both metrics.

**Figure 9:**
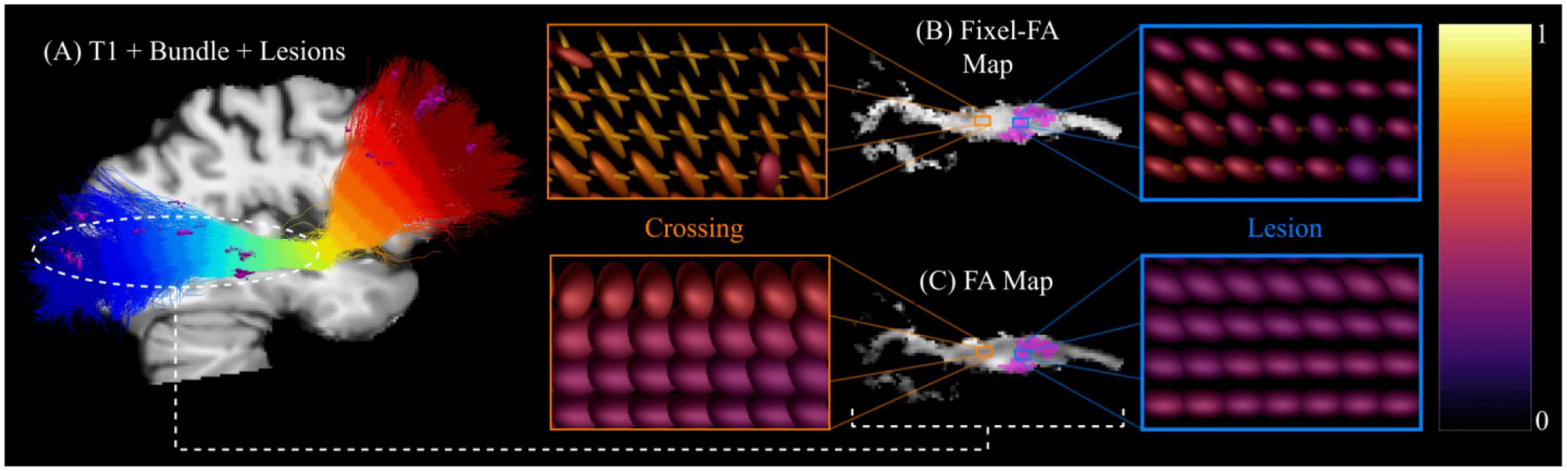
Difference between DTI and MTM in ROIs with crossing fibers and lesions. **(A)** The T1 image of an MS patient and the IFOF_L bundle streamlines with lesion mask (pink colored surfaces) are shown for anatomical reference. **(B)** Fixel-FA and **(C)** FA maps along the IFOF_L bundle estimated with MRDS and DTI, respectively. Tensors and multi-tensors are rendered in two different ROIs using the FA value of the tensor as color using the Inferno color map. One of the ROIs (blue ROI) has MS lesions, while the other (orange ROI) is located at the NAWM. Both ROIs present crossing fibers as the IFOF intersects other bundles. Single-tensors show a decreased FA value in both ROIs. Multi-tensors show low FA value only in the ROI with lesions, while the ROI in the NAWM shows more consistent values with healthy tissue.

Figure 9 displays FA and fixel-FA maps along the IFOF_L bundle in the patient 004. Both single- and multi-tensors are visualized in the region of the bundle. Each tensor is colored according to its FA value. Additionally, Figure 9 compares the tensor renders in two different ROIs within the bundle. The ROI outlined in blue has MS lesions while the orange outlined ROI is located at the normal-appearing white matter. Several crossing fibers are present in each ROI as the IFOF bundle crosses with other bundles, such as the PYT and ILF bundles. The FA map shows darker areas in both ROIs, corresponding with the shape, and decreased FA showed by the tensors. No significant differences in FA values are appreciated between the two ROIs. On the other side, the fixel-FA map is darker only in the ROI with the lesion. However, unlike the FA map, fixel-FA map shows higher values and fewer dark spots in the crossing fiber ROI. This indicates that fixel-FA is more robust to crossing fibers. In addition, multi-tensors show FA values within a healthy control range in the crossing fiber ROI, highlighting their potential to differentiate between crossing fibers and lesions.

## 4 Discussion

In this work, we address the crossing fiber bias from DTI metrics used in tractometry, by instead using multi-tensor fixel-based measures obtained from multi-shell HARDI acquisitions. Multiple b-vale diffusion-weighted data is mandatory for reliable parameter estimation in MTM-based methods such as MRDS (Scherrer and Warfield, 2012; Coronado-Leija et al., 2017). Our multi-shell acquisitions remain clinically feasible (~30 minutes).

Previous works in literature have reported limitations when informing tractometry with multi-tensor fixel-based metrics. Multi-tensor fitting is computationally demanding, highly affected by noise, and requires extensive high-quality dMRI HARDI acquisitions, which are time-consuming and challenging to find in clinical settings (Tournier et al., 2011; Jeurissen et al., 2013). Because of this, previous tractometry methods informed with multi-tensor fixel-based metrics have been limited up to 2 fixels per voxels, resulting insufficient in many regions of the brain (Delinte et al., 2023; Mishra et al., 2014). MTM methods generally struggle to accurately determine the number of fixels at each voxel, which is especially challenging in regions with complex fiber configurations. Choosing MRDS as a framework to estimate the multi-tensor fixel-based measures and using TODI to inform MRDS's model selection with tractography regularization allowed us to address these limitations in the current state-of-the-art. MRDS accounts for the presence of up to 3 fixels within each voxel plus an isotropic compartment, allowing for more accurate characterization of the fixel-specific tract profiles and being robust to fiber-crossing. MRDS is relatively fast when estimating the diffusivities in the resampled WM at 1 mm isotropic resolution (~1 h of computing time per subject). Moreover, it has been shown to be accurate and robust to noise (SNR = 12) when using clinical-grade dMRI data and protocols. Finally, the new model selection (Karaman et al., 2023) applied in MRDS allows for improvement in the estimation of the required number of tensors per voxel, taking advantage of the spatial regularization provided by tractography.

In the experiments with synthetic dMRI data, relative errors for fixel-based metrics indicate that multi-tensor fixel-based metrics estimated with MRDS are robust to crossing fibers and sensitive to WM anomalies (Figure 5). When comparing the tract profiles obtained with fixel-based multi-tensor metrics to traditional single-fixel tensor metrics, a difference in sensitivity was observed. Tract profiles informed with multi-tensor fixel-based metrics distinguish between crossing fibers and scenarios like axonal loss and demyelination (Figure 6) by assessing the underlying fiber configuration and WM tissue metrics.

We tested our proposed tractometry pipeline on several WM bundles of the *in-vivo* healthy control group: SLF, AF, CG, IFOF, PYT, ILF and MCP. We compared the obtained tract profiles informed with multi-tensor fixel-based metrics with tract profiles informed with single-tensor metrics along these bundles, focusing on their robustness to crossing fibers (Figure 7). The robustness of the multi-tensor fixel-based metrics to crossing fibers is evident across all examined bundles. Besides, our findings indicate that tractometry informed with multi-tensor fixel-based metrics is consistent, reliable, and not significantly affected by random noise or crossing fibers. As expected, single-tensor metrics exhibit a notable fluctuation when the estimated number of crossing fibers per voxel (*N*) along the bundle increases or decreases. This pattern suggests that single-tensor metrics are highly influenced by crossing fibers. According to the literature (Grieve et al., 2007), tract profiles informed with multi-tensor fixel-based metrics exhibit FA values in a range that is considered normal for healthy WM. This alignment suggests that multi-tensor fixel-based metrics provide more accurate representation of the WM integrity. Contrary, singles tensor metrics fail to estimate FA values considered normal in the WM because they are biased by crossing fibers.

In Section 3.2 we quantitatively and qualitatively explored the within-subject and between-subject variability of the tract profiles. The consistent low SDs values for the tract profiles indicate minimal variability within and between subjects. Despite the higher variability in multi-tensor fixel-based tract profiles, they remain within acceptable limits. This suggests that multi-tensor fixel-based informed tract profiles are more accurate, but less precise than DTI informed tract profiles. Moreover, we conducted an ANOVA test to evaluate the differences in mean fixel-FA and fixel-RD metrics across 20 locations of several bundles in sub-015. The overall rejection rates across the labels suggest a high level of consistency in the measurements, with an average rejection rate of 40%. However, our findings indicate that the tract profiles of certain bundles are significantly influenced by the anatomical location, revealing significant differences in the means of fixel-FA and fixel-RD across different regions of the brain. These results underscore the importance for careful interpretation of tract profiles as certain bundles, particularly in subjects with pronounced variability.

While Rojas-Vite et al. (2019) provided a solid foundation for the application of fixel-based metrics provided by MRDS, further validation using animal models remains essential. Particularly, in the context of demyelination and tractometry. Understanding the intricate changes in the obtained fixel-based metrics associated with demyelination is crucial for accurately interpreting the alterations detected by our method. Future studies utilizing animal models have to be driven for a more comprehensive assessment of our approach's sensitivity to demyelination and its correlation with histological outcomes.

### 4.1 Application to relapsing-remitting multiple sclerosis

We compared relapsing-remitting MS patients to a group of healthy subjects with similar age and brain configuration (Figure 8). The proposed pipeline shows to be sensitive to WM anomalies related to relapsing-remitting MS disease. The single MS patient tract profiles exhibit values that clearly deviate from the healthy control group. These deviations are potentially related to MS pathology as they occur around lesion location. A similar behavior is reproduced in the synthetic data simulating demyelination (Figure 6). Therefore, differences between group-averaged and individual MS patients' tract profiles in Figure 8 are assumed to be a consequence of the disease. In general, for all bundles, MS patients consistently show reduced fixel-FA and increased fixel-RD compared to healthy controls.

In Section 3.3, we made a comparison between the tract profiles of the HC group and two MS patients (sub-004-ms and sub-022-ms) of the MS group. Although a group comparison (HC vs. MS) may be done, the inherent group-averaging may not be beneficial because of the variability of MS lesions among MS patients. MS lesions can appear in different regions along the brain, and the severity of these lesions varies between patients (Wicks et al., 1992). Averaging these tract profiles across patients could lead to loss of critical information that is essential for understanding the individual differences within the MS group. Nonetheless, it is important, as a future work, to design an analysis for the entire MS cohort, which will provide a more comprehensive understanding of these dynamics. Moreover, we recognize the importance of developing a framework for explicit comparison of Wallerian degeneration, which would provide valuable insights to the MS research community. Finally, we acknowledge the need for a more comprehensive analysis comparing FA and RD values in the normal-appearing white matter of MS patients with those of healthy controls, which could further enhance our understanding of the integrity of WM in regions without visible lesions.

In a previous study (Winter et al., 2021), tractometry with dMRI metrics was investigated in young adults with relapsing-remitting MS. They reported significant abnormalities in the WM microstructure in WM bundles similar to those we used. In particular, reduced FA and increased RD were observed, indicating demyelination, which aligns with our reported results. Additionally, specific changes in fiber density and complexity were noted, indicating axonal degeneration. In Chamberland et al. (2021) a study using tractometry was conducted on MS patients with optic neuritis. It was found a limited ability to differentiate between various types of lesions like demyelination and axon loss using dMRI metrics, which is consistent with our findings. In another example (Beaudoin et al., 2021), tractometry informed with single tensor and other advanced fixel-based metrics was used to investigate the association between diffusion MRI-derived measures and neuropsychological symptoms of MS. They focused on WM fascicles that are associated with cognitive dysfunction in the presence of lesions. Our approach could offer several benefits to this kind of studies. For example, MTM metrics may replace standard DTI metrics in their analysis. The integration of these new metrics should be direct, as MTM metrics have the same biological and geometrical interpretation as DTI metrics without the crossing fiber bias. This could provide a more robust and accurate depiction of microstructural WM changes in MS patients. MTM metrics like fixel-RD would allow for a more precise and sensitive characterization of demyelination and other alterations, including axon loss. Robust multi-tensor metrics could improve the reliability of longitudinal studies by providing consistent and accurate measures over time. This would facilitate the monitoring of disease progression. By incorporating multi-tensor fixel-based tractometry analysis, researchers and clinicians may underscore the advantages of multi-tensor fixel-based metrics in improving the fidelity of studies.

One of the main limitations in the current literature is that RD metric can be contaminated in regions with crossing fibers and lesions, leading to erroneous interpretations and conclusions, making RD unstable as a biomarker (Jones et al., 2013; Winston, 2012). This work addresses this limitation by offering a tractometry pipeline robust to crossing fibers, suggesting the fixel-RD metric as a more robust biomarker for demyelination. Our pipeline shows that multi-tensor fixel-based methods could be a robust alternative to DTI, in which familiar metrics such as FA or RD are now specific to a particular fixel or track, with similar biological/geometrical interpretation. This facilitates the contextualization of these MTM metrics regarding many studies utilizing DTI metrics. Besides, it is unnecessary to include other fixel-based metrics such as AFD, which has challenging biological interpretability. AFD reflects the density of axonal fibers within a voxel, but not necessarily their functional status or health (Dell'Acqua et al., 2007). Thus, an increase or decrease in AFD does not directly translate to improved or deteriorated neurological function, requiring additional context (Raffelt et al., 2012). Moreover, pathological conditions like demyelination or axon loss can alter diffusion properties in ways that are not straightforward to disentangle, making it hard to pinpoint the exact biological cause of changes in AFD (Jones et al., 2013).

### 4.2 Limitations

In this work, we utilized a simulated phantom that incorporates different compartments to simulate WM microstructure to evaluate our proposed method. However, it is important to acknowledge the limitations of this phantom as it only serves as an approximation that does not capture the full complexities of the human WM. Membrane permeability and vascularization are examples of factors that were not considered in these simulations. Future work should focus on validating the proposed method using more realistic phantoms, such as the proposed by Callaghan et al. (2020) and Villarreal-Haro et al. (2023).

Our pipeline uses the closest-fixel-only strategy when relating the streamline's segments to local fixel properties. This does not allow multiple local fixels to contribute to a given streamline and might contribute to erroneous tractometry results if the bundle does not have enough streamlines. This can be improved by employing a fixel angular weighting strategy as the one proposed and used in Delinte et al. (2023).

Our results showed a decrease in RD precision when a single fiber population is present. This is concordant with what has been reported in other multi-fiber methods (Parker et al., 2013). Including a free water tensor in MRDS enhances results accuracy and mitigates potential biases, particularly when analyzing patient data. Nonetheless, this inclusion decreases sensitivity in the estimated fixel-based diffusivities due to the increased complexity of fitting 4 tensors (3 anisotropic and 1 isotropic) instead of 3 with MRDS. Besides, for acquisition schemes including high b-values the estimation of *N* and the isotropic volume fraction is affected, see Appendix A. Hence, the isotropic compartment may partially explain the contribution of the extra-cellular part of the dMRI signal, resulting in a reduction of the RD as shown in results with synthetic data. In the future, we consider that it will be important to study in depth the impact of including a free water compartment in MRDS and their implications in other lesions like edema as it is still an open question.

In our study, we demonstrated that the proposed method effectively detects variations in tract profiles associated with lesions, both in synthetic simulations and *in-vivo* data. This capability underscores the potential of our approach for identifying abnormalities in complex fiber crossing regions. However, it is important to note that while our method shows promise in detecting lesions, future work is necessary to further investigate its performance in accurately assessing the actual severity of detected lesions.

Although the obtained results underscore the capabilities of the proposed pipeline to identify WM lesions while being robust to crossing fibers, it cannot discriminate between demyelination and axonal damage. This is congruent to previous studies, which found that RD is sensitive to several microstructural changes different from demyelination, such as axonal deterioration, edema, and inflammation (de Vries, 2010). More advanced models like SM (Ferizi et al., 2016; Novikov et al., 2019) can distinguish between changes occasioned by axonal integrity and changes due to demyelination, but they still use one single tissue kernel per voxel, not per fixel. Similar to Dayan et al. (2015), our robust multi-tensor fixel-based metrics can be combined with these advanced methods, leading to a more sophisticated pipeline with a different type of metrics. Additionally, the employment of **M**agnetization **T**ransfer **I**maging (MTI), which is sensitive to myelin content, could help to differentiate between demyelination and axonal injury. However, it is necessary to extend the developed phantom for simulating not only dMRI acquisitions but also MTI acquisitions in order to validate the results on *in-vivo* data.

Another important aspect to consider is the high amount of false-positive streamlines in the tractogram and recognized bundles (Maier-Hein et al., 2017). While segmenting the tractogram and focusing the analysis on known tract bundles, false-positive streamlines can lead to inconsistencies in the tract profiles of the tractometry analysis, like overestimating the tract profiles from the estimated fixel-based metrics. Additionally, they can introduce more noise and variability into the analysis, hindering reproducibility. This can reduce the sensitivity of tractometry analysis to detect genuine alterations in WM between control subjects and patients, resulting in misinterpretations and erroneous conclusions. Fortunately, there are methods like COMMIT (Daducci et al., 2015) that assign weights to individual streamlines in the tractogram by solving a convex optimization problem. This enables the detection of false-positive streamlines, which can be removed by discarding streamlines with weight equal to 0. As future work, COMMIT can be integrated into the pipeline to obtain a pipeline more robust to false-positive streamlines.

## 5 Conclusions

In conclusion, our work focuses on creating a robust tractometry framework informed by tractography-regularized multi-tensor fixel-based metrics. It demonstrates its capabilities to address the crossing fibers bias and lesions, increasing the sensibility in both simulated and real-world scenarios.

This study makes several key contributions to the field of WM imaging analysis. First, developing a simulated phantom with challenging and customizable geometry, incorporating different WM scenarios by using the standard model (healthy tissue, demyelination, and axon loss). This phantom provides a controlled environment to systematically evaluate and compare different imaging techniques and models. This allows us to verify the accuracy and robustness of our proposed methods against various fiber configurations and pathologies. Second, our proposed pipeline informed with the multi-compartment framework MRDS–three anisotropic and one isotropic compartment–marks a substantial methodological advancement. This pipeline goes from raw data to tract profiles informed with track-specific tensor metrics. By combining tractography robust to lesions and accurate multi-tensor fixel-based metrics, our pipeline achieves more robust, precise, and sensitive representations of the WM microstructure, particularly in regions with complex crossing fiber configurations or lesions related to pathologies. This approach addresses limitations in the current state-of-the-art methods. Thirdly, we evaluated the proposed tractometry pipeline in a cohort of 20 healthy individuals. Our results demonstrate the superiority of MTM over DTI, highlighting MTM's enhanced ability to capture detailed microstructural information and resolve crossing fiber geometries. The increased sensitivity of MTM metrics provides more accurate assessments of white matter integrity. Finally, applying our tractometry pipeline to a cohort with relapsing-remitting MS further underscores the clinical relevance of our work. Our qualitative analysis demonstrates the sensitivity of the pipeline in detecting WM anomalies related to demyelination. This is particularly important in diseases like MS, where it is important to differentiate between crossing fibers and lesion contamination. Pipeline's capabilities to delineate these anomalies offer an improvement over those that only include DTI metrics for studying and monitoring MS and potentially other neurological conditions.

## Figures and Tables

**Figure A1:**
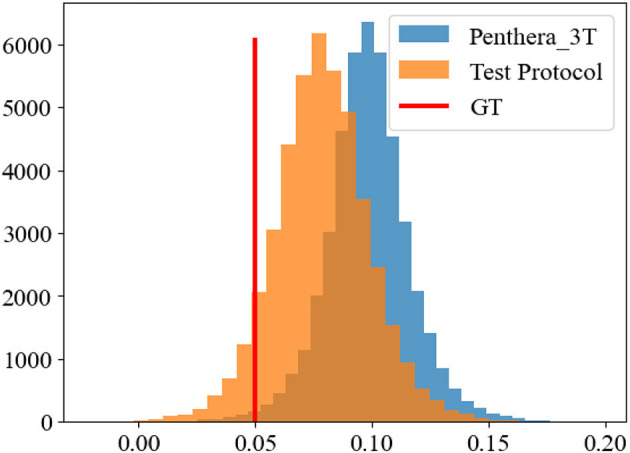
Histograms of the estimated isotropic volume fraction with MRDS using different protocols.

## Data Availability

The raw *in-vivo* dMRI data and the derivatives are not accessible to the public as we do not hold exclusive ownership of the data. Nevertheless, data can be solicited with a reasonable request and data-sharing agreements with the owners. A Python framework was developed to simulate the synthetic phantom's diffusion-weighted imaging (DWI) signal. The code can be found in https://github.com/ErickHernandezGutierrez/phantom_inferno. The Tractometry_flow pipeline adapted to the MRDS fixel-based metrics can be found in https://github.com/scilus/tractometry_flow. The visualizer used to render the tensors and multi-tensors is available at https://github.com/scilus/dmri-explorer. The *Tractoflow* pipeline is available at https://github.com/scilus/tractoflow.

## Appendix A

### Isotropic compartment fitting

Two synthetic datasets were generated using 2 different acquisition schemes. Datasets share the same parameters except for the scheme. They were generated with no noise and no dispersion. MRDS was fitted in both datasets in order to study the estimation of the **I**sotropic **V**olume **F**raction (IVF) with different schemes. The first scheme (Penthera_3T) matched as closely as possible the scheme used in the *in-vivo* experiments. Similar to the Penthera_3T scheme, the second scheme (Test scheme) was composed of 107 encoding directions, but they were distributed as 33 directions for *b* = 0.3ms/μm^2^, 33 for *b* = 0.6ms/μm^2^, 34 for *b* = 1ms/μm^2^, and 7 for *b* = 0, respectively.

Figure A1 shows histograms of the estimated IVF with MRDS on the 2 different acquisition schemes. The histogram corresponding to Penthera_3T shows an overestimation of the IVF, with a mean relative error of 80%. On the other hand, the histogram corresponding to the second scheme has a lower relative error (40%). This indicates the importance of using an appropriated protocol for the estimation, as the free diffusion compartment decays faster for high b-values (>1ms/μm^2^), reducing the number of lower b-values measurements in the protocol biases the estimation of the IVF. However, for the most accurate estimation of the free water compartment acquisitions with multiple echo times are needed (Coelho et al., 2022).

## Appendix B

### Fiber tracking with multi-tensor

If a statistical-based model selection, such as the Bayesian Information Criterion (BIC), is used to obtain a preliminary MTF with MRDS, an **O**rientation **D**istribution **F**unction (ODF) can be generated. The transformation of the MTF provided by MRDS into an ODF image can be performed using the following equation (Daducci et al., 2014):

(B.1)
$$\begin{array}{l} ODF\left(\hat{g}\right)=\overset{N}{\underset{j=1}{\sum }}\alpha_{j}\frac{{\left({\hat{g}}^{T}D_{j}^{-1}\hat{g}\right)}^{-\frac{3}{2}}}{4\pi \sqrt{det\left(D_{j}\right)}}, \end{array}$$

Fiber tracking can be performed using the probabilistic tractography algorithm iFOD2 (Tournier et al., 2009) on the ODFs obtained with Equation B.1. Since iFOD2 has been optimized for the **F**iber **O**rientation **D**istributions (FODs) derived from CSD, a tuned ODF amplitude cutoff of 0.05 should be used as it has shown to improve results when tacking with multi-tensor derived ODFs (Girard et al., 2023). Afterward, the obtained tractogram can be used as input for the proposed pipeline in Figure 4. Thus, this tractogram can be segmented into major bundles, employing RecoBundlesX. Besides, it can be used to evaluate a new MTF with the TODI model selector.

Computing the tractogram with MRDS instead of CSD could help to reduce the pipeline's computational time. However, both RecoBundlesX and iFOD2 are specifically tuned to work with ODFs obtained with CSD or similar methods. Therefore, the quality of the tractogram may be affected, resulting in difficulties recovering some major bundles.

## Appendix C

### Tract profile standard deviation and ANOVA test results for fixel-FA and fixel-RD metrics

**Table C1:** Standard deviations (SD) for tract profiles across different white matter bundles.

	**SLF_L**		**AF_L**		**PYT_L**		**ILF_L**		**MCP**		**IFOF_L**		**CG_L**	
**Subject**	**FA**	**Fixel-FA**	**FA**	**Fixel-FA**	**FA**	**Fixel-FA**	**FA**	**Fixel-FA**	**FA**	**Fixel-FA**	**FA**	**Fixel-FA**	**FA**	**Fixel-FA**
sub-003	0.108	0.174	0.117	0.142	0.122	0.143	0.112	0.167	0.098	0.213	0.113	0.154	0.116	0.170
sub-004	0.113	0.158	0.113	0.164	0.127	0.161	0.102	0.167	0.105	0.177	0.108	0.163	0.111	0.174
sub-005	0.118	0.154	0.103	0.160	0.136	0.170	0.100	0.172	0.120	0.192	0.105	0.165	0.118	0.148
sub-006	0.110	0.162	0.112	0.167	0.126	0.163	0.102	0.170	0.108	0.179	0.103	0.165	0.119	0.173
sub-007	0.101	0.172	0.099	0.153	0.128	0.153	0.104	0.163	0.112	0.162	0.106	0.154	0.109	0.160
sub-008	0.115	0.169	0.102	0.151	0.135	0.172	0.091	0.158	0.106	0.170	0.102	0.143	0.112	0.168
sub-010	0.108	0.159	0.100	0.145	0.123	0.162	0.093	0.158	0.112	0.196	0.099	0.153	0.100	0.161
sub-011	0.099	0.162	0.110	0.162	0.123	0.146	0.103	0.156	0.116	0.185	0.100	0.146	0.132	0.168
sub-012	0.102	0.192	0.106	0.162	0.135	0.174	0.108	0.158	0.121	0.182	0.115	0.155	0.111	0.186
sub-014	0.105	0.172	0.100	0.152	0.129	0.167	0.092	0.173	0.102	0.163	0.098	0.170	0.093	0.159
sub-015	0.109	0.144	0.108	0.142	0.120	0.165	0.109	0.180	0.101	0.208	0.103	0.171	0.116	0.161
sub-016	0.112	0.154	0.104	0.146	0.130	0.173	0.098	0.155	0.107	0.190	0.102	0.144	0.126	0.152
sub-018	0.114	0.175	0.103	0.167	0.130	0.156	0.108	0.169	0.107	0.198	0.113	0.159	0.121	0.156
sub-019	0.114	0.171	0.104	0.146	0.128	0.171	0.110	0.153	0.106	0.161	0.117	0.151	0.105	0.170
sub-020	0.100	0.156	0.094	0.135	0.128	0.169	0.099	0.163	0.109	0.203	0.103	0.157	0.109	0.162
sub-021	0.121	0.171	0.108	0.155	0.126	0.150	0.105	0.175	0.110	0.206	0.103	0.164	0.117	0.167
sub-022	0.108	0.161	0.103	0.142	0.123	0.166	0.105	0.153	0.113	0.223	0.103	0.150	0.106	0.164
sub-023	0.108	0.178	0.099	0.129	0.130	0.158	0.098	0.163	0.109	0.200	0.110	0.152	0.119	0.173
Cohort	0.109	0.166	0.105	0.151	0.128	0.162	0.102	0.164	0.109	0.190	0.106	0.157	0.114	0.165
**Subject**	**RD**	**Fixel-RD**	**RD**	**Fixel-RD**	**RD**	**Fixel-RD**	**RD**	**Fixel-RD**	**RD**	**Fixel-RD**	**RD**	**Fixel-RD**	**RD**	**Fixel-RD**
sub-003	0.507	0.346	0.521	0.355	0.538	0.378	0.503	0.341	0.452	0.307	0.478	0.352	0.525	0.346
sub-004	0.491	0.357	0.491	0.373	0.527	0.367	0.483	0.348	0.498	0.333	0.489	0.369	0.512	0.337
sub-005	0.483	0.354	0.473	0.370	0.543	0.386	0.464	0.342	0.508	0.344	0.485	0.371	0.522	0.358
sub-006	0.507	0.371	0.487	0.385	0.536	0.384	0.476	0.352	0.506	0.339	0.486	0.366	0.505	0.337
sub-007	0.476	0.339	0.471	0.342	0.540	0.374	0.472	0.333	0.491	0.315	0.478	0.347	0.497	0.320
sub-008	0.493	0.352	0.479	0.340	0.536	0.365	0.474	0.347	0.496	0.320	0.465	0.349	0.518	0.328
sub-010	0.471	0.341	0.457	0.332	0.527	0.369	0.461	0.337	0.502	0.323	0.456	0.338	0.489	0.321
sub-011	0.475	0.352	0.486	0.365	0.524	0.366	0.472	0.336	0.507	0.318	0.481	0.345	0.510	0.326
sub-012	0.488	0.368	0.474	0.370	0.545	0.388	0.482	0.340	0.518	0.329	0.477	0.349	0.496	0.358
sub-014	0.496	0.351	0.479	0.364	0.532	0.378	0.458	0.341	0.477	0.309	0.485	0.361	0.487	0.329
sub-015	0.478	0.365	0.491	0.368	0.537	0.371	0.474	0.336	0.504	0.319	0.480	0.366	0.507	0.345
sub-016	0.505	0.354	0.473	0.363	0.543	0.376	0.464	0.342	0.491	0.326	0.469	0.342	0.515	0.345
sub-018	0.473	0.351	0.477	0.362	0.538	0.386	0.486	0.339	0.500	0.324	0.478	0.361	0.519	0.332
sub-019	0.501	0.357	0.489	0.360	0.546	0.374	0.485	0.337	0.496	0.317	0.490	0.345	0.507	0.337
sub-020	0.484	0.365	0.467	0.348	0.545	0.381	0.465	0.338	0.506	0.330	0.469	0.343	0.492	0.343
sub-021	0.474	0.341	0.484	0.349	0.539	0.357	0.467	0.332	0.488	0.319	0.477	0.348	0.522	0.319
sub-022	0.472	0.351	0.469	0.345	0.538	0.371	0.471	0.330	0.494	0.312	0.473	0.348	0.504	0.317
sub-023	0.487	0.353	0.465	0.331	0.542	0.384	0.470	0.337	0.504	0.327	0.481	0.341	0.511	0.345
Cohort	0.489	0.355	0.478	0.360	0.539	0.376	0.474	0.339	0.497	0.324	0.479	0.352	0.505	0.340

The SDs for each bundle are calculated by taking the square root of the mean of the within-subject variance across five repeated scans. The last row in the table shows the SD obtained from the between-subject variance, reflecting the variability of the tract profiles within the group. The SDs are reported for four key metrics: FA, fixel-FA, RD and fixel-RD. Only 18 of the 20 subjects from the HC cohort are reported because 2 of them did not completed the five scans.

**Table C2:** ANOVA results for tract profiles of sub-015 across the 20 sections (labels) of multiple bundles.

**Fixel-FA**	**SLF_L**		**AF_L**		**PYT_L**		**ILF_L**		**MCP**		**IFOF_L**		**CG_L**	
**Label**	**f-stat**	**p-value**	**f-stat**	**p-value**	**f-stat**	**p-value**	**f-stat**	**p-value**	**f-stat**	**p-value**	**f-stat**	**p-value**	**f-stat**	**p-value**
1	2.68	6.20E-01	1.95	2.20E-01	13.45	1.26E-08	2.54	6.98E-01	4.60	3.13E-02	1.13	2.33E-01	0.40	5.42E-01
2	0.52	1.51E-01	1.64	8.66E-01	3.96	3.84E-04	1.64	1.69E-02	0.77	1.21E-03	3.59	2.13E-02	9.33	5.24E-07
3	1.81	7.20E-01	2.01	1.17E-02	8.50	6.73E-03	6.15	3.93E-02	2.23	3.36E-02	1.41	3.03E-02	2.63	1.94E-02
4	1.68	1.16E-01	2.67	8.57E-01	0.17	5.55E-01	0.39	4.31E-04	3.45	5.91E-01	1.70	7.32E-01	8.92	3.88E-02
5	0.42	2.10E-01	2.28	2.18E-02	3.74	3.96E-04	0.88	6.89E-01	7.52	5.83E-07	1.33	7.70E-01	4.16	7.90E-04
6	1.83	1.37E-01	2.20	5.03E-01	4.20	2.57E-01	9.50	1.43E-02	0.27	8.34E-02	0.98	2.23E-01	5.27	1.89E-05
7	1.73	1.31E-01	5.35	2.41E-04	22.91	5.45E-17	13.83	1.88E-13	6.97	1.88E-05	0.74	1.36E-01	1.29	1.36E-02
8	4.63	2.74E-04	2.04	1.26E-01	14.76	1.05E-06	15.27	2.82E-06	5.12	1.77E-02	8.89	1.71E-05	2.82	1.45E-01
9	4.10	5.86E-03	4.43	5.58E-05	4.00	5.20E-03	2.47	7.17E-03	2.20	3.60E-02	5.87	1.97E-03	3.25	3.33E-03
10	1.39	1.12E-01	5.14	1.33E-03	2.01	1.19E-01	5.13	3.66E-01	7.64	9.55E-03	3.57	5.01E-04	3.73	1.12E-03
11	1.03	9.90E-01	0.54	2.68E-01	1.93	1.47E-02	3.38	2.62E-01	1.83	2.70E-01	3.40	6.62E-04	2.68	6.22E-05
12	1.81	4.28E-02	12.99	5.45E-07	10.16	1.48E-07	13.85	1.67E-05	4.59	2.87E-03	5.61	3.61E-01	0.36	9.03E-01
13	4.87	8.56E-03	7.40	3.10E-04	7.12	1.83E-01	9.42	1.79E-06	4.68	6.92E-05	6.23	1.15E-04	0.60	4.13E-01
14	2.54	9.77E-03	4.50	1.47E-01	3.66	4.24E-03	3.18	2.61E-05	5.15	6.68E-01	1.33	4.30E-01	13.63	1.85E-05
15	0.78	5.48E-02	4.10	3.25E-02	3.79	1.66E-02	1.97	1.12E-01	1.07	3.41E-01	11.86	1.63E-03	5.78	1.11E-05
16	9.05	3.11E-03	18.82	3.47E-13	5.31	1.63E-02	0.65	4.07E-01	2.87	4.70E-02	8.91	1.27E-05	5.50	1.28E-04
17	2.10	4.94E-01	7.85	5.87E-05	4.41	2.98E-02	3.37	2.59E-01	9.96	2.63E-06	7.06	2.39E-02	8.27	8.73E-09
18	0.51	2.61E-01	0.69	2.25E-01	10.31	3.31E-06	6.81	3.43E-09	1.13	2.93E-01	17.16	1.87E-09	4.89	2.39E-03
19	1.81	2.80E-01	1.51	3.20E-01	2.18	1.06E-01	0.99	6.23E-01	3.77	4.23E-01	1.99	2.02E-01	0.22	1.43E-02
20	2.73	4.56E-01	0.57	1.66E-01	2.19	3.92E-02	6.77	1.90E-02	1.82	8.15E-01	1.92	1.38E-02	0.98	7.60E-01
Rej. rate	25%		35%		50%		40%		35%		40%		55%	
1	2.55	3.65E-01	0.87	9.90E-02	7.81	2.08E-04	1.47	4.42E-01	1.78	3.29E-02	2.13	2.78E-02	3.84	8.35E-02
2	0.99	1.58E-01	1.88	6.29E-01	0.85	3.83E-01	2.42	1.84E-02	2.89	1.65E-02	2.34	3.10E-01	7.45	1.37E-04
3	1.94	2.11E-01	1.09	4.50E-01	6.21	4.59E-04	1.52	2.19E-01	0.34	7.36E-03	1.49	6.28E-01	2.10	8.33E-02
4	1.30	7.04E-02	1.19	5.44E-02	13.20	2.87E-05	1.91	4.24E-01	8.90	5.56E-05	1.30	2.52E-01	1.66	9.10E-02
5	0.86	1.56E-01	0.95	3.13E-02	19.43	1.46E-10	1.46	9.16E-01	0.95	5.26E-01	1.13	4.03E-02	2.30	3.59E-01
6	1.98	3.94E-02	11.33	6.11E-07	18.96	1.45E-10	2.85	3.57E-01	8.11	3.62E-07	6.99	1.79E-03	6.71	1.19E-05
7	2.40	3.11E-01	6.17	2.27E-06	8.92	1.40E-06	4.00	1.41E-05	16.90	4.07E-09	1.30	7.30E-01	7.27	8.59E-04
8	0.33	4.04E-01	3.50	1.90E-01	3.60	1.14E-02	0.21	6.85E-01	3.24	1.62E-04	2.08	2.70E-01	2.24	9.10E-02
9	1.04	5.36E-01	10.65	4.61E-08	1.06	1.14E-02	0.62	2.15E-02	3.34	1.44E-02	3.52	1.06E-01	3.22	2.18E-03
10	0.00	7.94E-06	7.05	2.33E-03	8.26	1.20E-03	3.21	3.24E-01	26.34	3.35E-16	11.59	5.33E-10	3.51	4.32E-02
11	0.47	1.64E-01	9.52	4.65E-07	2.17	5.06E-03	1.06	7.70E-01	12.96	7.62E-10	12.14	4.52E-08	5.19	8.09E-02
12	0.00	3.13E-07	4.51	1.98E-01	3.05	7.44E-03	4.01	1.25E-01	2.94	1.66E-01	6.51	1.92E-06	9.12	1.71E-06
13	4.57	1.28E-02	4.98	3.98E-02	0.47	1.34E-01	3.17	3.03E-01	2.10	2.18E-03	9.48	1.58E-04	6.49	1.41E-02
14	4.02	1.55E-03	3.60	3.18E-03	6.92	2.12E-01	0.30	3.22E-01	1.71	1.22E-01	0.70	8.55E-01	2.97	7.49E-03
15	3.78	7.62E-03	10.43	1.04E-04	0.92	7.24E-03	3.75	1.43E-05	1.45	3.62E-01	4.72	4.24E-02	3.65	2.98E-02
16	0.44	1.46E-01	7.04	4.07E-07	6.83	7.45E-06	3.47	8.95E-03	3.18	2.68E-06	4.99	4.21E-03	6.23	3.71E-05
17	2.33	2.43E-02	1.87	1.00E-01	1.01	7.09E-03	0.57	3.25E-01	2.58	4.11E-03	1.22	3.28E-01	2.66	1.94E-01
18	11.29	1.80E-05	1.26	8.85E-01	2.18	2.96E-02	3.04	1.21E-03	11.60	1.45E-06	1.41	2.01E-03	7.80	4.88E-03
19	2.88	2.88E-02	2.55	3.54E-01	6.76	4.35E-02	2.41	6.38E-03	14.73	1.13E-07	0.75	2.52E-01	1.03	8.64E-02
20	1.91	5.04E-01	1.55	1.84E-01	2.64	1.00E-05	4.66	2.01E-03	1.41	7.36E-03	1.32	7.63E-02	4.20	8.93E-02
Rej. rate	25%		40%		60%		25%		60%		25%		40%	

The ANOVA test assesses whether there are statistically significant differences in the means tract profiles with fixel-FA and fixel-RD metrics across locations, with a significance threshold set at *p* < 0.01. It includes the percentage of hypothesis rejection. The sample size for the analysis is 500.
